# *“You Think You’re Helping Them, But They’re Helping You Too”*: Experiences of Scottish Male Young Offenders Participating in a Dog Training Program

**DOI:** 10.3390/ijerph14080945

**Published:** 2017-08-22

**Authors:** Rebecca J. Leonardi, Hannah M. Buchanan-Smith, Gill McIvor, Sarah-Jane Vick

**Affiliations:** 1 Psychology, Faculty of Natural Sciences, University of Stirling, Stirling FK9 4LA, Scotland, UK; h.m.buchanan-smith@stir.ac.uk (H.M.B.-S.); sarah-jane.vick@stir.ac.uk (S.-J.V.); 2 Scottish Centre for Crime and Justice Research, Faculty of Social Sciences, University of Stirling, Stirling FK9 4LA, Scotland, UK; gillian.mcivor@stir.ac.uk

**Keywords:** animal-assisted intervention, dog training program, prison, young offenders, dogs, human-animal interaction

## Abstract

Interaction with animals can be beneficial to humans and animal-assisted interventions (AAIs) are increasingly popular in a range of contexts. Dog training programs (DTPs) are the most popular form of AAI in custodial contexts; prisoners often have multiple needs and DTPs seem to facilitate a diverse range of positive outcomes, including improvements in well-being, behavior, and offending behavior. However, evidence on the efficacy of prison-based DTPs is still limited and these evaluations often lack detail or methodological rigor. We examined the experiences of male young offenders (N = 70) using thematic analysis of semi-structured interviews conducted following completion of a DTP. The themes that emerged indicated a broad range of inter-related experiences and positive outcomes. The most prevalent theme related to their experiences with Dogs (including feelings and attitudes), and there were perceived improvements categorized as: Positive Effects (including mood and well-being), Motivation, Charitable Purpose, Self-Efficacy, Improved Skills, Impulsivity, and Emotional Management. These themes mapped well onto outcomes previously identified in research on DTPs, and to the program’s core aims of improving behavior, educational engagement, employability, and well-being. The diversity and nature of these themes indicates that DTPs have considerable potential to engage and benefit those individuals with multiple needs, such as young offenders, and ultimately to achieve positive long-term outcomes with significant social, health, and economic impact.

## 1. Introduction

Interactions with animals are associated with a wide range of physical and psychological benefits for humans, and are particularly effective in enhancing interpersonal communication and reducing stress and anxiety [1,2,3,4]. Animal-assisted interventions (AAIs) recognize the role that animals may serve as catalysts or mediators of human social interaction, assisting in therapeutic processes by simultaneously relaxing and engaging the client [5,6]. AAI is increasingly being used in a range of contexts including custodial settings [7,8,9]. Although there is a diverse range of prison-based animal programs, dog training programs (DTPs) are considered particularly effective and these have increased in popularity in recent years [9,10,11]. There is considerable variation in DTP programs between facilities but the most common types are a community service model, preparing rescue shelter dogs for rehoming, and a service animal model, providing socialization as preparation for advanced assistance dog training [7,9,12]. DTPs differ from most AAIs with other populations because the human-animal interaction is not solely for the therapeutic benefit of the prisoner, or used in conjunction with clinical therapy methods [7,10]. Moreover, the interaction usually extends to providing care and training for the dogs, and many programs also include vocational or educational components to enhance employability [7,10,12,13,14,15].

Arluke [16] has proposed that through their experiences training dogs, participants discover and practice positive new ways of thinking, feeling, and behaving. Participants gain personal insights and increased self-esteem through the achievement of targets and goals with the dogs [17,18,19]. This enhances participants’ personal development, by increasing problem-solving abilities and empathy, encouraging teamwork, enhancing social skills, and recognizing the positive impact of prosocial behavior [15,16,17,18]. For young offenders, and particularly those who suffered emotional and physical abuse from caregivers, canine companions also provide social comfort and help to re-instill trust and confidence in others [15,16,17,20]. The opportunity to participate in animal programs can be an incentive for inmates to improve behavior; in combination with improved engagement and skills this has the potential to contribute to a reduction in recidivism [21]. Perceived benefits are not limited to program participants; for example, other prisoners, staff, and community members are also positive in their overall evaluations of DTPs [13,17,22]. In community service programs, the effective rehabilitation and reintroduction of the dogs into the community delivers additional positive outcomes, by both improving their welfare and ameliorating a societal problem in dealing with unwanted animals [12]. The process of allowing inmates and animals to help each other toward mutual rehabilitation, while also benefiting the wider community, has been described as a potential win-win-win situation [7].

Quantitative evaluations have used a range of research designs and outcome measures, including clinical symptomatology and psychological functioning, and institutional and re-offending behavior [9,11]. In terms of psychological wellbeing, DTPs have been reported to improve scores on self-esteem and depression scales [13,23], reduce loneliness, and improve self-efficacy, interpersonal control, and enhanced relationships in female prisoners [13]. Fournier et al. [21] identified improvements for male prisoners in terms of self-reported progression in therapeutic treatment, a social skills inventory, and reduced criminal behavior as indicted by the number of institutional infractions. A recent review identified 10 evaluations of DTPs that used quantitative outcome measures but six of these were unpublished or commissioned reports [9]. Overall, the meta-analyses indicated positive but small effect sizes for changes in psychological well-being. However, several methodological issues, including heterogeneity of interventions, research designs and measures implemented, constrain the interpretation of these findings [9,11,24]. In addition, these evaluations are typically based on small samples that are positively biased by the stringent selection processes for program participation [7,8].

In contrast, qualitative research describes and interprets how and why an intervention effects change within a given context, generating rich data regarding the experiences of participants and identifying key mechanisms for change. Understanding the underlying processes can enhance the efficacy of an intervention, generate hypotheses, and underpin the choice and interpretation of quantitative outcome measures [24]. Although there are numerous anecdotal descriptions of the perceptions and outcomes of DTPs in custodial contexts, there remains a paucity of systematic qualitative research, and even fewer studies that triangulate qualitative and quantitative measures [9,11]. Moreover, these reports are not all peer reviewed publications, and often do not adhere to relevant standards for contextualizing qualitative research, which recommend the provision of adequate methodological details, and a consideration of issues relating to interpretation, such as theoretical orientation, researcher reflexivity, cross checking, grounding in examples, and attention to negative cases [25,26].

Turner [27] used in-depth unstructured interviews with six adult male offenders participating in a service animal DTP (USA) to gain insight into participants’ experiences and perceived benefits. Cross-case content and thematic analysis identified seven key themes that described the beneficial outcomes for participants: patience; parenting skills; helping others; increased self-esteem; social skills; normalizing effect; calming effect on the environment. However, strict selection criteria were in place for the program, and the report does not specify any theoretical orientation, provide reflection by the researcher, or consider any negative aspects of the participants’ experiences identified in the data [25,26]. Davis [14] used structured interviews to examine the experiences of male young offenders (N = 14) currently participating in a vocational community service model (USA). Detailed contextual information is lacking in this unpublished report, but content analysis and frequency of response types were used to identify five themes: patience and responsibility, developing a relationship with the dogs, work skills of learning and teaching, communication/social skills, and technical skills. Furst [28] explored the implications of the relationships that develop between prisoners and dogs, in terms of developing a prosocial sense of self. Participants in two different DTPs (USA; 15 female and seven male prisoners) reported improvements in patience, feeling a sense of accomplishment from their achievements with the dogs, improvements in communication, facilitation of relationships (including with their families), and providing opportunities to help others. These factors are considered to contribute to the development of a prosocial identity, and increase the likelihood of future desistance from crime [28,29]. However, the methodology was not well described, including lack of detail regarding interview procedures, and the methodological reflections were limited, making it difficult to assess the contribution of the research.

Currie’s [15] unpublished report provides a more comprehensive evaluation of a service animal program (USA), which conforms closely to recommended qualitative guidelines and considers the program from five perspectives: male inmate trainers (N = 16), former trainers (N = 6), other inmates (N= 3), staff members (N = 5), and the researcher’s reflections on the program and evaluation process. Reported positive emotional outcomes were: positive social support; sense of pride gained; increased patience; improvement in self-esteem; feeling of giving back to society; humanizing element and connection to the outside world. Positive practical outcomes were: improvement in responsibility; more positive prison environment; opportunities to help others; goal setting and achievement; employability skills gained; motivation and improvement in behavior. The positive emotional and practical outcomes reported from multiple perspectives are congruent with common themes identified in participants’ self-reports in other studies [14,27]. Negative aspects of the program were also identified, including the responsibility required of inmate trainers, emotional difficulties in giving up their dogs, and potential for conflict with other inmates.

Despite the prevalence of DTPs in the USA and Canada, these programs have not received comparable uptake in the UK and this seems due, at least in part, to a reluctance to adopt this model without an evaluation of efficacy within the context of the UK prison system. Although there is a long history of prison animal programs in the UK [19,30], including a few institutions that have resident dogs on site [31], the program evaluated here (Paws for Progress) is the first DTP, introduced at HM Young Offenders Institution Polmont (Scotland) in 2011. This DTP was developed using the “5 Step Approach”, as advocated within the Scottish criminal justice system. In this framework, reviewing the evidence base and ongoing processes of evaluation are integral to program initiation, development, and delivery; this approach not only documents whether a program is effective, but aims to identify how and why, and under which conditions [32,33].

Young offenders in custody represent a high-risk, vulnerable population with disproportionately high rates of unmet physical, developmental, social, and mental health needs, and higher mortality [34,35]. The social adversities experienced by young male offenders prior to conviction are frequently related to difficult family backgrounds and a lack of social support [36,37,38]. Psychological distress and suicide risk are high, particularly for those from socially disadvantaged backgrounds [37,39,40]. The impact of social disadvantages on psychological distress may be intensified by deficits in social problem-solving skills [41]. Individuals with the most impoverished interpersonal skills may be most vulnerable to the stresses of incarceration, including higher suicide risk [41,42]. The risk of reoffending is higher in those with the greatest need for cognitive skills interventions and lower employability skills [43]. This population represents a challenge in terms of social determinants of health, such as poverty and educational attainment [44], and rehabilitation has significant implications for public health outcomes, including mental health, addiction, and injury and violence prevention [45,46]. Offenders have multiple needs and interventions tackling a range of problems are considered the most effective [32,33]. A range of positive outcomes across several domains have been identified for DTPs, using both qualitative and quantitative methods of evaluation [7,9,11], which suggests that DTPs may be particularly effective in addressing the multiple needs of offenders [34,35,47]. However, the evaluation of prison animal programs is challenging because of the constraints imposed by an applied context, in which multiple individual and program variables potentially confound results [20,21]. Research findings are often difficult to contextualize [25] because these programs are diverse in nature and there is often insufficient detail provided on participant or program characteristics [7,8,9,11,24].

The identification of key areas of need and anticipated change in previous research informed both the development and evaluation of the DTP [24,32,33], in relation to the four key aims of the program: to improve behavior, to increase educational engagement, to develop employability skills and to enhance well-being. While mixed methods (qualitative and quantitative) were used in parallel to evaluate the program overall [24,48], the current study examines the underlying processes and intermediate outcomes using thematic analysis of semi-structured, post-course interviews with program participants. Qualitative methods are particularly useful for identifying similar patterns across interventions [24] and given the common components of DTPs the themes identified are expected to overlap with previous findings. Qualitative research indicates that prison-based DTPs are associated with a broad range of positive effects [9,14,15,27,28] but samples are often small and may not be representative because of comprehensive screening in the selection of participants, limiting conclusions on those benefits directly attributable to the program [9,24]. The current study addresses these issues, with a larger sample recruited in the absence of strict program selection criteria, allowing for an evaluation of both the prevalence and diversity of participants’ experiences.

## 2. Materials and Methods

*Study Site:* HM YOI Polmont is Scotland’s national facility for male young adults (16 to 21 years) either awaiting trial or are convicted young offenders (serving all sentence lengths). The most common sentence lengths are between two and four years [49]. Over the evaluation period, HM YOI Polmont held approximately 350–700 convicted young (≤21 years) male inmates.

*Paws for Progress Intervention:* Paws for Progress is a Community Service DTP for male young offenders, in which positive reinforcement training is used to prepare rescue dogs for rehoming. The program was designed to be mutually beneficial; it aims to improve the behavior and welfare of participating dogs, and to improve behavior, increase engagement in education, develop employability skills, and enhance wellbeing of the young men. The duration of the course is 8-week, with a maximum of 10 young men participating in a session (6 new recruits and 4 returning as assistants/peer mentors). Although there were changes to the program (particularly in resourcing) following the pilot phase (two courses), the basic course design and delivery remained consistent. Each student trainer is paired with a dog and their work is focused towards helping the dog be rehomed. Participants learn how to train and care for the dogs; they design training plans and use positive reinforcement methods to achieve their training goals. Rescue dogs are present in 3 sessions per week, with dogs belonging to staff ordinarily present in other sessions. The sessions take place in dedicated indoor and outdoor dog training areas. During non-practical sessions, participants learn the theory behind dog training and animal care and complete coursework. Each course also includes visiting speakers from a variety of organizations.

*Participants:* Seventy male young offenders (16–21 years) completed the program over the evaluation period (12 courses, July 2011–August 2014), only four participants did not complete the program (all due to transferal or release). Sixty-six (94%) completed interviews immediately following course completion. Sixty-four participants progressed to course assistant or mentoring roles and 11 (17.2%) completed interviews while in these roles.

Institutional records were used to examine program participant characteristics (additional detail is provided in Tables S1–S3). In summary, 54% of participants had not previously served a custodial sentence, 21% had served one, and 24% had previously served two or more. At least 61% of participants experienced significant adversity during their childhood, including being raised in care, suffering trauma, childhood neglect or abuse, and significant bereavement. In terms of mental health, 40% of participants had records indicating a history of issues and almost all (93%) had a history of substance abuse. Approximately half of the participants (47%) had no previous qualifications prior to beginning Paws for Progress. Only a quarter (26%) had experienced previous regular employment (i.e., >1 month) and 50% had no previous work experience of any kind. Over half (57%) had not previously engaged with any courses or learning support available through the YOI.

*Recruitment:* Participation in the program and evaluation processes was voluntary and the program was advertised across the institution. Candidates’ ability to participate in the intervention program was based on their availability and sufficient time to complete course. Selection for the program was never conditional upon prior good behavior, or precluded based on criminal offence type [7]. Informed consent was gained for participation in the course and evaluation. The study was conducted in accordance with the Declaration of Helsinki, and the protocol was approved by the Psychology Ethics Committee, University of Stirling, and the Scottish Prison Service Ethics Committee. The dogs were recruited from local dog shelters (Dogs Trust: West Calder and Glasgow Rehoming Centres, Scotland), and transported to the HMYOI Polmont for training sessions. Dogs and inmates were always under supervision to ensure appropriate welfare standards were maintained.

*Procedure:* One-to-one semi-structured interviews were conducted in a quiet area of the workshop, or in an interview room at the YOI. Interviews were audio recorded (Casio Dictaphone). Questions were impartial and non-leading and the same to all participants, with additional prompts used as needed (see Table S4 for interview schedule). The interview focused on participants’ experiences on the DTP, including how enjoyable and useful they had found it, any changes they felt had resulted from participation, their experiences of working in groups, any comparisons to previous learning experiences, their experiences working with the dogs, and any recommendations to improve the course. Interviews ranged between 1 min 35 s and 18 min 23 s in duration. In addition to post-course and peer-mentor interviews, written statements were voluntarily provided by 23 participants (including 5 participants following release or transferal) and these were included in a single document, resulting in 78 sources in total.

*Data manipulation and analysis:* Audio files from all interviews were transcribed and analyzed in NVivo 10 (QRS International, Melbourne, Australia). Transcriptions were coded by interview question before themes were created as nodes to which responses could be coded. Similar themes were independently identified by a research assistant (V.K.) for 12 interviews, with differences only in terminology used, indicating reliability. As the analyses progressed, thematic categories could be restructured; themes were also examined for internal convergence and external divergence (i.e., internally consistent but distinct). Themes were not mutually exclusive and responses could be assigned to multiple themes. NVivo generates frequency score for sources assigned to each theme, and quotations are used to ground the interpretation [25].

## 3. Results

In terms of responses to specific interview questions, all participants gave positive responses when asked about their enjoyment of the DTP course, experiences with the dogs, and experiences working in a group. All participants felt that the course had been useful and all but one perceived change in themselves (see Table S5 for more detail on responses according to question). The frequencies of themes across participants in the initial post participation interviews (N = 66) are provided in Table 1 (see Table S6 for frequencies across all sources). These themes will be explored in order of descending frequency, theme labels are in bold and capitalized and sub-themes in bold. Finally, negative experiences are also considered. Quotations are italicized and indented and are identified by participant number (P1–P70) with source type (PI = post-course interview; PMI = peer mentor interview; WS = written statement; additional examples are provided in Table S7).

**Dogs:** Participants were asked directly about their experiences with the dogs during the course; many participants discussed their feelings towards the dogs (97%), changes in attitudes towards dogs (95%) and dog training (88%) at some length. Their experiences with dogs were integral to their experiences on the program more broadly, as evidenced by the inter-relatedness of this theme to others described below, for example, in terms of Charitable Purpose in helping the dogs towards being rehomed. Around half of the participants (48%) also identified parallels between themselves and the dogs, in terms of their previous experiences and perceived change.

**Feelings about dogs:** Almost all participants described strong positive emotional experiences in relation to their interactions with the dogs:

“The personality of my dog was fantastic. I love the dog to bits. I enjoyed every day I was up here cos I knew I was going to see the dog. Got to bond with the dog. Gave him a wee bit more comfort. Taught him better skills. Great.”

(P57:PI)

The positive relationships they developed with the dogs is well encapsulated in several descriptions of mixed emotional responses when a dog was rehomed:

“Sad to see them go man, but it’s only for the best isn’t it? I felt happy in myself as well aye. For doing the work for them and that. Know what I mean? It does feel good, feel really proud of yourself.”

(P21:PI)

**Attitudes to dogs:** Almost all participants described their positive attitudes towards dogs, and several also indicated that this had not always been the case. Some participants described feeling increased empathy and emphasized the need for dogs to be treated with respect:

“When I used to have dogs, I’ve had dogs all my life. If the dog was bad I’d raise my hand to it so it would get scared and it wouldn’t do it again. I’ve realized that that doesn’t work. All that does is builds up and builds up and builds up and you do it again and it just wants to bite you. I never realized that before, I just thought if you make it scared then it’ll be submissive and you’re the leader of the pack. But positive reinforcement, treats, toys or whatever. They feel comfortable working for, so… That’s what I’ve learned, that you can’t just raise your hand to a dog. I just thought it’s a pet. Since working here I’ve realized that you can’t treat dogs like the way I was treating them.”

(P40:PI)

**Attitudes to dog training:** Attitudes to training were frequently discussed, with an emphasis on what they had learned about the effective use of positive reinforcement methods:

“You think, you get them to do things by getting angry and giving them a row. But we’re learning, we’re learning it’s not the best way. We’re learning to think about how we can ignore things, not reward it if you don’t want it… (the behavior)… walk away from it if it gets too much. Give it a rest for a few minutes and then go back to it, make it easier and try again. I’d have never done it, thinking about training as rewarding in that way.”

(P10:PI)

Participants often indicated their willingness to share their knowledge with others:

“I’m just going to basically teach people what to do with their dogs and that. Just give them a bit of a heads up—you’re maybe doing this wrong but you can do it, do these wee steps to make it better. So I think it’s a good advantage and I’m really happy with what I’ve been doing.”

(P33:PI)

**Parallels with dogs:** Almost half the participants drew parallels between the experiences and changes in themselves and the dogs, highlighting a specific strength of these type of programs:

“I think coming down to the dogs is really good. It made me feel good about myself, knowing I am helping a dog and I feel more calm knowing I’ve got my dogs as a friend. And not just rehabilitating to dogs, I think I’m getting rehabilitated as well from my point of view.”

(P60:WS)

**Positive Effects:** Almost all participants attributed positive effects to the program and related these to their own enjoyment (98%), positive changes to the prison environment (91%), the therapeutic nature of the program including bonding with the dogs (52%), and improved mood and wellbeing (30%).

**Enjoyment:** Almost all participants related their enjoyment of the program to their positive experiences and engagement, often enhanced by the dogs’ mutual enjoyment of their interactions:

“Aye it’s been brilliant, aye, it’s a good laugh and that as well. I enjoyed the obstacle courses and that. Doing that, it’s a good laugh and that with the other boys and the dogs really enjoy it so, aye.”

(P17:PI)

For some participants, the variety of the activities included contributed to the enjoyment:

“Paws for Progress is the best work party in Polmont because every day is different and it is really enjoyable.”

(P28:WS)

Positive social interactions were often considered as important for enjoyment, including mutual respect and descriptions of working with others as productive:

“It’s been good working in the group too, it’s a laugh at times, we enjoy it but really, everyone gets on, everyone works hard.”

(P12:PI)

Several participants identified enjoyment as integral to their learning and longer-term engagement:

“I think Paws for Progress is the best course. It’s helped me to work as a team and stay calm and out of trouble. In addition the staff are very good and helpful towards us. I enjoy the course and so I hope to stay on and increase my skills even more.”

(P64:WS)

**Change from institutionalized/prison environment:** The opportunity to participate in a DTP was not what many had expected in this environment:

“I think the course is perfect. I enjoy it very much. Just getting to come down and spend time with dogs and that. It’s not really what you thought you’d get to do in prison.”

(P49:PI)

The consistent opportunities to learn, make progress and achieve across a range of activities was perceived as being distinct from other activities available:

“Naw, it’s good. It’s alright, you’re not sitting listening to somebody talking. Or just sitting writing or that. You’re just doing a bit of everything, getting skills, achieving stuff.”

(P43:PI)

Similarly, some felt that the course was more engaging than the alternatives available:

“Aye, I’ve enjoyed it. It’s the best. It’s the only thing I’ve liked, that I’ve stuck with. I’d do anything to be kept on it too.”

(P8:PI)

The presence of the dogs was also perceived as normalizing the institutional environment, and providing a sense of freedom despite the custodial security:

“I think it’s a good work party to have because it gives you a wee bit of a sense, not of freedom as such, but just to walk about with the dog and that. Gives you a sense of being outside again without actual being on the other side of the wall.”

(P33:PI)

Some participants valued the sense of autonomy and independence gained through experiences on the course, which developed the skills and confidence needed to approach new environments:

“It’s much better than all of that (other work parties). Working with the dogs, thinking for your self—it’s good, it’s good that way, working out what to do for them yourself. It’s a bit of freedom. A feeling of what it’s like out there. Some people have been banged up for ages and we’re not used to that.”

(P11:PI)

**Therapeutic effects including bonding:** Although this course is an AAI and not an Animal Assisted Therapy (AAT), because it was not designed to work to a therapeutic goal or directly tackle offending behavior, some participants did draw parallels between the effects of participating on this course with prison programs and therapy more broadly:

“It’s serious but at the same time you get a good laugh and that when you’re down here. When you’re doing programs and that it’s serious, you’re always, there’s no time for relaxing and all that. You’re always like, dead serious all the time. But down here it’s a more relaxed environment. You can enjoy it at the same time while you’re working.”

(P32:PI)

Several participants recognized the positive effects of human-animal interaction on general atmosphere and social connections, and in relation to opportunities for caring and affection reciprocated by the dogs:

“I just enjoy it so I do. I enjoy seeing the dogs and that. Like seeing them running about, happy and that in here, cos they get on with people dead easily. Jet as well, he’s a good dog, he cares about people. I’ve really enjoyed what I’ve been doing.”

(P33:PI)

Interacting with the dogs was also considered to be relaxing and calming, both socially and in providing opportunities to enjoy one to one interactions:

“I enjoyed it best in the kennel—in the booths. Getting your own one to one time, no distractions. Just basically bonding with the dog.”

(P30:PI)

Some participants identified a sense of developing a positive identity, a mutually beneficial bond, and mutual rehabilitation as a result:

“The dogs—they change the way you think and the way you act. They trust you, it’s that mutual bond. You think you’re helping them but they’re helping you too.”

(P6:PI)

**Improved mood and wellbeing:** Positive mood was commonly associated with being around the dogs and was enhanced by the pleasure and satisfaction in observing positive changes:

“Aye, makes me happy, just basically being around the dogs and that. Just noticing the difference you’ve made from the first day you meet the dog until when the dog leaves. Just the gradual steps involved. You start to notice them. You feel good about it as well. You feel happy with yourself. That you’re teaching an animal how to do the basics.”

(P33:PI)

Several participants described how they felt that the course was a constructive use of their time and skills, providing a positive focus in an otherwise stressful environment:

“It’s given me something really constructive to do with my time as well. I think that without this course I would’ve been lost. So it has, it’s been a wee bit of a god send as well.”

(P4:PMI)

A few participants were referred directly to the program by prison staff because they were finding the prison environment particularly challenging; as one of these individuals explains, being around the dogs enhanced their mood and provided a sense of respite from their situation:

“And, I like working with dogs, I’ve always liked animals. I like being with the dogs. It makes me feel happier being down here. When I get down here, it makes me feel good. You know, much better than I did before.”

(P7:PI)

**Motivation:** Almost all participants (95%) expressed high levels of motivation, and this was explained by their enthusiasm for the program (91%), to the rewarding nature of the work (78%) and a sense of commitment and responsibility (56%).

**Enthusiasm:** Almost all participants clearly expressed how engaged and motivated they were by their experiences, frequently offering multiple explanations rather than singling out any one aspect. When asked to compare their experiences to those on other programs, most felt it was the best available, and many seemed unable to recommend any changes to improve the course:

“It’s the best it can be. It’s phenomenal. Helped a lot of dogs. Helped a lot of people. Everything’s up to scratch.”

(P57:PI)

Some participants were excited by their own achievements:

“See, like I was saying before, I would NEVER have known about how to click and treat a dog, or help it follow your hand, like that to sit. Stuff like that, it’s just (notices the dog next to him sat at his signal)—good girl, see stuff like that! I do it without even realizing now! That’s brilliant.”

(P7:PI)

Others felt inspired by the benefits gained by all those involved in the program:

“I think Paws for Progress is a great project to be a part of. It gives prisoners good opportunities to get qualifications and certificates. It also gives the dogs that take part a better chance of getting rehomed. It’s a work party that I personally enjoy coming to, I think this project should be an on-going thing!”

(P54:WS)

Several participants described their experiences as being transformational:

“Paws for Progress has been excellent for me as I have learned lots of new skills and have gained qualifications. I now know when a dog is stressed and scared. I was also very impatient before I started but now I am patient as you should be with the dogs. It has been a life changing experience for me and I love working with the dogs.”

(P30:WS)

**Rewarding:** Most participants discussed the rewarding nature of their experiences. Some perceived a direct relationship between their efforts and rewarding returns with the dogs; they felt that while they were helping the dogs, the dogs gave them more back in return:

“And if you give her even a wee bit of time, she gives you a lot more back. Show her that you care and that you’re paying attention to her, and she’ll do whatever you want her to do. Just need to be patient with her. She’s a lovely dog, she’s brilliant. Wouldn’t change her for anything.”

(P12:PI)

Others felt rewarded by the sense of autonomy gained from teaching:

“When you know they never used to be able to do that, and knowing that you’ve been able to train them to do it—that’s what I liked best. Like Buddy. He’s come on a lot since I started working with him, a lot better.”

(P37:PI)

Similarly, personal development gains were considered rewarding, with some participants also relating this to a more positive self-appraisal:

“Paws for Progress has helped me to become a better person by understanding the way dogs think and the way they act around other people and dogs. And it feels like a real achievement when the dogs get rehomed.”

(P55:WS)

**Commitment and responsibility:** Motivated principally by their desire to help the dogs, participants frequently reported being surprised at how hard they were working, how committed they were to their responsibilities, and how they felt they had progressed personally as a result:

“I just thought I changed a lot more than I’d have thought. I’ve definitely changed. I’ve matured a lot more, more responsible, aye.”

(P1:PI)

Some participants felt their commitment to the course was distinct from their previous experiences with other activities:

“It’s better than anything else here, I think. It’s the only thing I enjoy, that I look forward to coming to. Everything else you’re like, ugh, I need to go down there—but this, you’re up and ready, waiting to come down first thing in the morning.”

(P10:PI)

Most participants were keen to progress to a mentoring role, indicating high levels of commitment; for some this was related to a desire to gain additional responsibilities, perceived as beneficial when returning to the community:

“It’s like the upcoming peer mentors, who will take over from me—they’ve got a lot to offer as well. It would be good to give them a wee chance at a bit of responsibility as well, you know, before they get out.”

(P4:PMI)

While high levels of social enjoyment were clearly important for many participants, this was often balanced by a shared sense of responsibility towards the dogs:

“It’s good, it’s a good laugh. Loads of banter as well. But, I think like, you see a change in the boys. When the dogs come up, there’s a kind of seriousness. Like we all go out and do what we do. I think everybody on the course knows how to be, working with dogs.”

(P40:PI)

A clear sense of ownership of the project and a collective responsibility to ‘make it work’ were also described by a few participants, for example, in relation to opportunities to demonstrate their own abilities and the program’s achievements:

“When visitors came in and all that, to see what we’re doing. It’s always been alright. See when the visitors come in, there’s never been one thing that’s gone wrong. The dogs are always alright and we’re always alright. Show what we can do, what we can achieve.”

(P38:PMI)

**Charitable Purpose:** The charitable purpose of their work was evidently important and most participants (85%) referred positively to the opportunity to benefit others, either in relation to teaching (70%) or helping others (65%).

**Teaching others:** As trainers participants were simultaneously teachers and learners, an experience which some clearly valued and enjoyed. Many participants gained satisfaction from teaching the dogs; a process which served to validate their efforts as they were not only teaching effectively but also using these skills for a worthwhile purpose:

“Just seeing Laurence coming in the way he was and then the way he’s leaving. He’s learned a lot. I’ve trained him, taught him a lot. I enjoy seeing that I’ve got something to give to a dog.”

(P36:PI)

Some participants expressed that they were also keen to use their skills to teach others, including peers and their families:

“I’m really looking forward to teaching my dogs, using the stuff I’ve learnt down here to teach them to calm down. Especially so it works now I have my wee man when I get back out. And I feel more like I can teach my wean about it too.”

(P11:PI)

For some participants, the best aspect of the program was the sense of accomplishment gained from teaching others and sharing in their achievements:

“Even apart from the qualifications and certificates we get at Paws for Progress, we get something more than that. It’s the sense of accomplishment, when you’ve taught something, even just one thing. Whether you’ve taught your dog or you’ve helped another student, you feel like you achieved a real goal. And when my dog achieves something, well then I’m really happy, because I know that dog is one step closer to getting a home.”

(P40:PI)

**Helping others:** The non-confrontational social dynamics within sessions, combined with the positive response of the dogs to interaction and affection, provided an outlet for caring and helping others. The realization that they could ‘make a difference’ was frequently emphasized by participants as integral to their positive experiences. In most cases, seeing the dogs make progress seemed to outweigh the participants’ own progress:

“I enjoy it all basically. The bit I enjoy the most is like, not just me but when the dog achieves something. When the dog passes his APDT Good Companion Award. If he completes that I’ll be happy. When the dog achieves something it’s one step closer to getting a home.”

(P57:PI)

Some participants clearly recognized that interactions with the dogs were mutually beneficial:

“I would say Paws for Progress is a very successful project as it gives prisoners a second chance to gain some qualifications and also gives the dogs a better chance of getting rehomed.”

(P30:PI)

When describing the impact on the dogs, participants sometimes went beyond their own direct involvement in training and considered their general welfare and prospects for the future:

“The dogs that are on the course also benefit greatly from taking part. They receive one to one training and get to socialize with people and other dogs more than they would normally staying at the rehoming center. And due to the stimulation they get from being on the course, this helps make them less stressed while back at their kennels. All of which improves their chances of being successfully rehomed so everyone involved in the training program is a winner.”

(P28:PI)

Several participants stated their commitment to help others in other contexts, and were passionate about goals that they perceived to be worthwhile:

“I enjoyed collecting donations for the dog charities as I wanted to give back to them and dogs, for all the help we’d been given. After finishing the training course, for the first time in my life I knew what I wanted to do with the rest of it—work with and help animals.”

(P8:WS)

For several participants, helping others was considered important in terms of personal development:

“It’s good to see the change in the dog. But when you first get your dog, it doesn’t know nothing, then when you work with it you can see progress and all that. It’s not easy but it’s good. It’s good to see that you’re helping a dog that’s came from nothing. I’ve never done nothing to help anybody in my life, so when I do this it makes me feel alright cos I’m doing something good. When I don’t usually.”

(P38:PI)

‘Giving’ as a form of reparation was also identified as potentially playing an important role in rehabilitation, as described by a peer mentor:

“It’s gonna to be good, constructive stuff for folk, and that is important. Cos people are happy, they’re happy to come to it, it’s something they will really appreciate and it will be fulfilling for them, to do something positive. A lot of the things people do, it’s purely for the parole board, ticking boxes. But imagine how that is for people serving long sentences. They’ve got nothing to work for. This would be really constructive for them, and something they want to do.”

(P4:PMI)

**Self-Efficacy:** Self-efficacy, a belief in one’s ability to succeed in specific situations, plays a major role in how an individual approaches goals, tasks, and challenges [50]. Most participants reported improved self-efficacy (88%), in terms of a sense of achievement (70%), enhanced self-confidence (56%), sense of autonomy (38%), problem-solving abilities (30%), or aspirations for the future (30%).

**Sense of achievement:** Many participants expressed the sense of achievement they gained from observing changes in their dog’s behavior, and awareness that successfully rehomed dogs had a happier future. The belief that they had something positive to offer, having developed the ability to teach and provide help to others, was valued as a worthwhile accomplishment.

“Aye I’ve really enjoyed it so I have. I felt that I’ve made a difference with the dogs if you know what I mean. Like, I’ve already rehomed 3 dogs so it’s been a good experience and that. Quite pleased with what I’ve done. Feels like I’ve accomplished something for a change, know what I mean? Just, good seeing the dogs leave. You feel happy when they’re away to a new home.”

(P33:PI)

Some participants also discussed their personal achievements in parallel with those of the dogs:

“I think that seeing how easy my dog changed his behavior had a huge positive impact on helping me change mine… My confidence also improved greatly during my time on the course and I think was largely due to the positive comments and praise I got for anything good that I done. Looking back now, I feel like a different person than the one I was when I arrived at Polmont, so thank you Paws for Progress for helping me to change and become a better person.”

(P8:WS)

**Confidence:** Through working together with the shared focus of helping the dogs, over half the participants felt that their interpersonal skills and confidence had improved:

“Aye, I’m more confident about like meeting new people, talking among a large group of people. Aye, my confidence is built up.”

(P24:PI)

Some of these participants considered how improved confidence was beneficial in terms of their motivation and ability to make the most of future opportunities:

“When I first came down to the course I was quiet, kept myself to myself but as the weeks went by I started to come out my shell and I was able to work as part of a group. Since leaving the prison I got help from Becca with doing my CV and I am now at college doing gardening. I would say that it gave me the confidence to go ahead and do this.”

(P41:WS)

**Sense of autonomy:** During the course, emphasis was placed on participants learning to set their own targets and assess progress. Some felt that this approach contrasted with their prior expectations and was felt to enhance enjoyment, a sense of ownership, and an appreciation of their achievements:

“I thought it was going to be things all lined up and do this and getting told what to do because that’s what the jail is all about. They just try and tell you what to do. But you do your own thing really with the dogs. You plan it yourself. You don’t just get told what to do and all that so. I didn’t expect it to be what it was going to be. I thought it was going to be worse than it actually is.”

(P18:PI)

The opportunity to take responsibility and help others, combined with the positive asset-based approach taken to skill development, was considered by some to facilitate engagement and foster confidence, particularly for those motivated to become peer mentors:

“It’s like me, see how I got so much out of this course, I think it’s cos, like a lot of folk in here, I’m a hands-on kind of person. Give me something to work with and I’ll do it. That’s the good thing with how you do things, you give us a chance to get on with it and we get stuck right in. I think you get to see people shine a lot more, when they progress to getting their own roles, working to their strengths—cos I think they’ll embrace that, getting a wee chance to do that their selves.”

(P4:PMI)

**Problem-solving:** Participants were clearly motivated to help the dogs improve their behavior and wellbeing. This process required them to assess each dog and plan an individual training program, which required setting targets and goals and measuring progress:

“I dunno, you want to help the dog because of what the dog’s been through and what could have been its past experiences. You want to give a bit back to the dogs. Focus on target and a goal. Like goal settings.”

(P57:PI)

Some participants highlighted importance of understanding each individual dog and building a positive trusting relationship:

“So it’s pretty much, both aren’t the same, but you can use one thing and it may work well with one dog, and you can try it with the other dog, but if they respond differently then you have to rethink. Once you get to know them, you work out what they like, and what they didn’t like.”

(P6:PI)

Others perceived the opportunity to translate from theory into practice as a valuable experience:

“The other stuff you just sit and talk about it, but with this you sit and talk about it then you actually go and do it. It’s much better.”

(P34:PI)

Some also recognized that the problem-solving skills gained, assessing situations and thinking carefully before addressing issues, could also be applied to other contexts with increased confidence:

“It’s good that way. Thinking about what you’re going to do and how you’re going to do it—it gives you this sense of freedom, solving the problem yourself.”

(P11:PI)

“Aye it’s good, thinking that way, aye. It’s helped us. To deal with other situations calmly. I think it has anyway.”

(P38:PI)

**Aspirations for the future:** Around a third of participants indicated that they were thinking more positively about the future, including potential employment or training opportunities following their release:

“I definitely want to follow it up when I get out. Hopefully there might be a chance for me to get involved in this, help dogs, it’s a project for me when I get out, and that will be brilliant.”

(P4:PI)

Some linked these aspirations to their own prospects, in terms of serving as positive role models for other young people, displaying a clear sense of pride in their achievements:

“Only that I hope I can contribute to this more again in the future… I think it would be good to come back, further down the line, be able to say to lads or folk that are doing it… you can say, I’ve been in your position, I know how it feels. But I’ve done something with this, made something of myself. So it’s all looking on the up from here.”

(P4:PMI)

Importantly, these aspirations were not always limited to themselves;

“It helps dogs be rehomed and they get better with training. I think Paws for Progress should keep going, so more dogs can be helped to find a good family. I think it should go to more jails, so more people can learn training skills and more dogs can be helped.”

(P42:WS)

**Improved Skills:** The majority (86%) of participants described improvements to their skills related to employability (64%) or education and learning (61%).

**Employment:** Many participants described improved confidence in their own abilities, and this was often related to their ability to work towards goals both independently and together as a team:

“Aye, it’s good, cos you are working as part of group and just yourself as well, so you get both experiences and skills, if you know what I mean.”

(P51:PMI)

Given the context of enjoyment and commitment to the program, the challenging nature of the work involved was perceived positively by some participants:

“I’ve found something that I’m good at, that I can work towards and use on the outside as well. But the peer mentor role here has been good as well for that, giving me a wee bit more responsibility as well, so it been good. It’s been a good wee challenge. I’ve fair enjoyed it.”

(P4:PMI)

Some participants identified the benefits of ongoing support provided by the program following release (including facilitating volunteering/work experience), in developing employability and aiding the transition from prison back to the community:

“Well this is the best one I’ve been on because I’ve been in hundreds of times, and every time I’ve got out nobody ever wants to help you but all the boys in here always get help when they go out. To do voluntary work eh, help with the CV building and all that stuff. So aye, better.”

(P70:PI)

For several participants, an awareness of previous participants’ successes following release served to enhance their motivation and self-efficacy, and provide a positive focus for the future:

“Aye definitely useful, it’s opened up doors for employment opportunities and self-employment, you know?”

(P28:PI)

**Education or learning:** Despite the frequently negative nature of their previous educational experiences, participants were generally enthusiastic about the learning opportunities facilitated. The variety and the relevance of their learning was important to some participants:

“Should be good to give other people advice about something I’ve learned in here. I never really thought coming in to the jail I’d learn and have an experience like this but I’ve actually quite enjoyed it for the simple fact that, working with the dogs and learning new things.”

(P60:PI)

Similarly, they were frequently enthusiastic the opportunity to learn from visiting speakers, and some felt this also provided an opportunity to demonstrate their own abilities to others:

“I think it actually gives folk a wee bit of a shock at first, when they see us differently, see what we actually can do. It’s like the folk who come in (external speakers), I don’t think they expect it at first, to get the kind of questions asked, to get the kind of focus and attention that we give them. Probably they expect to come in at first and think it’ll be a farce trying to teach us, but they haven’t had that. It’s been really, really good.”

(P6:PMI)

These participants often emphasized that enjoyment was central to their learning, highlighting the importance of embedding education within the context of their experiences with the dogs:

“Because this, you enjoy yourself while you do it even though we do a lot of work and eh, qualifications and that, we enjoy ourselves doing that, knowing that we’re going to be seeing the dogs and that after it too.”

(P69:PI)

Some participants described how their ability to learn had been enhanced:

“Aye, enjoying learning. It’s made it a lot easier and better for me. And I suppose then you could take skills from here and use it in other things.”

(P54:PI)

The positive approach to teaching on the course, which involved co-designing sessions, maintaining encouragement and flexibility to individual support needs, was considered important:

“You get more help. You can understand it more. You’re not getting told what to do, you’re getting explained how to be better at it. It’s not like shouting at you if you do something wrong. You just need to persist and keep practicing.”

(P67:PI)

Improved confidence in learning also facilitated subsequent engagement with education, as this participant who had been transferred to an adult prison explains:

“I have completed two creative writing modules, I’m currently working on my higher English with the view to starting Open University work in the summer and I have also just started a computing course. If it hadn’t been for Paws for Progress, I would never have even thought about attending education classes.”

(P8:WS)

**Social Impact:** Most program participants (83%) described positive effects on their social relationships; these were categorized as working together as a team (80%), peer support (52%), communication skills (33%), and family relationships including parenting (15%).

**Working together:** Most participants were positive in their assessment of the opportunities to work together, and some described their experiences with enthusiasm:

“It’s brilliant, I love the group, it’s like a perfect group. It’s brilliant, maybe it’s because it’s a good course. It’s really good being in the group.”

(P10:PI)

The dogs were commonly considered pivotal in helping participants relate to each other and encouraging the group to work together with a shared focus:

“Once the dogs are there everyone just gets on with it. You see the best side of people when they’re with their dog, and that makes it easier to talk to them.”

(P28:PI)

Many participants described improved interpersonal skills and the development of positive relationships. This was frequently identified as a distinguishing feature of the DTP, as many had previously struggled to relate to their peers and had felt isolated in the prison:

“It helps you with working in a group to build up relationships with boys and that. Know what I mean? Cos up the halls you don’t usually, you wouldn’t go and talk to a boy the way you do down here. ‘Cos we can get on with each other, work together, it’s a good group.”

(P38:PI)

This feeling of belonging to a positive social group was important to some individuals, and was related to their collective sense of responsibility and commitment to meeting the dogs’ needs:

“I don’t know, it’s a good atmosphere as well. I mean it’s not just working with the dogs, it’s a good laugh and that we have. Everybody gets on with everybody. Obviously we have a joke and a laugh and that and it’s all fun and games. But obviously when we get the dogs and harnesses on, everybody just gets his serious head on and we can do stuff. Get the head down.”

(P40:PMI)

**Peer support:** The importance of supporting each other to achieve their shared goal of helping the dogs was highlighted by some participants:

“You’re helping other people, doing the dog training, as well as helping the dogs. And it’s not just about helping your dog. You notice changes in other people’s dogs and you’re involved in that, noticing what they like and don’t like so everyone works together to make it easier for each other. So it’s like you’re helping them get better too.”

(P6:PI)

This included the importance of the support from peer mentors when joining the course:

“Yes, I liked working in the group, it was a lot of fun, and the people I’ve been working with were very helpful when I first started.”

(P9:PI)

The roles for continuing participants aimed to maximize opportunities for peer support, and some of these participants described skill development and sense of increased responsibilities:

“Working with Paws for Progress definitely helped me improve my team working skills, and encouraged me to help others. I felt really proud after I was asked to be a peer mentor—it felt like a big achievement.”

(P8:WS)

Some mentors related their skills in teaching others to their own experiences on the course, demonstrating compassion, empathy and understanding:

“Everyone here is right into it, that’s the thing, maybe it’s the course that does it. But it seems like everyone has just got stuck right into it, proper focused on it. And that’s the thing, I think most of these folk would be totally lost without it. Like look around you, how many of these guys have got stuck right into it, and it’s done a lot for them. It’s constructive—like look at (another student) doing so well, doing so well with it. Gives them something really good to look forward to, takes their mind off their sentence for a wee while. So it has been good, it’s been really good.”

(P4:PMI)

**Communication skills:** For a third of participants, learning to communicate effectively was important, and gaining the confidence to speak in front of others was commonly highlighted:

“I was quite quiet when I first came down but now that I’ve got to know people I’m speaking out a bit more. More confident.”

(P41:PI)

Although educational assessments were challenging for some, several participants reflected on the sense of achievement gained from engaging and improving communication skills:

“It’s helping me with reading and writing as well, getting to express myself in the writing, you know, the stuff I’m not saying, I get the chance to write all that down, which I find a lot easier now anyway. So it’s definitely been good.”

(P6:PI)

Effective listening was also identified as an important skill which facilitated working together and the development of positive relationships:

“Aye. Working in a group together was good, it was fun. Sometimes it was annoying at first, with other folk, too many people talking at once and you can’t get your ideas across. But once you learn how, and people listen to you, it’s much better. And then you’re much more able to listen to other people’s thoughts, sometimes they’ve got the same idea as you too, and then it doesn’t need to be difficult.”

(P6:PI)

Some participants felt that communicating more effectively also enhanced their self-confidence and subsequently their enjoyment of working together with their peers:

“I get on better with other people. Now I can put my point across a lot better. I feel more comfortable working with other people. Now it’s something that I am really good at, I enjoy it.”

(P10:PMI)

**Families and parenting:** Some participants reported sharing their experiences with family members, and described their desire to share their skills following release. The interest shown by family members facilitated positive conversations, and participants expressed a sense of achievement at committing to an activity which made their family proud:

“As well as helping me work with others as a team, being part of Paws for Progress brought me closer to my family too. It gave me something good to talk to them about, for a start. Then I felt like I was doing something to make them proud of me, the first time I could talk to them about something positive in years.”

(P8:PI)

Parenting skills were not specifically targeted within the course, however, a few participants made this connection themselves; describing how they would apply their understanding of positive approaches to leaning to interactions with their own children:

“It helps you think about things differently—I’ve got a wean, my wee man, he’s two. There’s a lot of it that’s the same—you learn not to give them a row, it’s better to encourage them for the good things, distract them away from doing something wrong. It’s helped me, think about how I can reward him. Instead of shouting at him for doing something wrong, reward him when he’s doing something right.”

(P11:PI)

**Impulsivity:** Over half the participants (58%) related their experiences to decreased impulsivity, a theme divided into improved behavior in the institution (45%) and self-control (36%).

**Institutional behavior:** Almost half of participants reported improvements in their behavior within the prison in general:

“Aye there’s no been any trouble since I started this. Aye, I like being down here with the dogs and that. When I wasn’t doing this dog training I used to get reports a LOT, know what I’m talking about?”

(P42:PI)

Participants were not threatened with removal because of misconduct reports in other areas of the prison, but several participants expressed concern about the potential consequences of their actions:

“It’s made me want to keep my head down. I want to stay on the course. If I get into trouble they’d probably take us off it.”

(P36:PI)

**Improved self-control:** Around a third of participants related their progress through the course with an improved ability to control their behavior and avoid conflicts with others:

“It teaches you different, it teaches you different as well. You learn not to solve things by shouting or threats or violence. It changes how you think about people, you think about why they’re acting the way they are.”

(P10:PI)

By considering the impact their behavior could have on others, participants were able to improve self-control; this was particularly prevalent in mentors’ responses:

“Paws for Progress has been a life changing experience for me. When I first came on to the course I didn’t like listening to people telling me what to do, but having the dogs there meant that to help teach them I had to listen. I have learnt to be more patient, to listen, and to understand dogs’ behavior and body language. Above all, I have learnt self-control, making me a better person.”

(P38:WS)

**Emotional Management:** Over half the participants (56%) described improvements in terms of patience (42%) or controlling anger (39%), which were not mutually exclusive but were distinct.

**Patience:** Many participants described improved patience in relation to the need to remain calm to be compassionate to the dogs’ needs, and to achieve training success by choosing an appropriate pace for each individual dog:

“Yeah I’ve been more relaxed and not nervous. You’re reacting differently, you’re patient. Instead of like telling it come on hurry up and do this, you are just relaxing and letting the dog go its own pace.”

(P57:PI)

Several described how improved patience was not restricted to their interactions with the dogs:

“It’s revealed how much patience I can have—for myself, for animals and for other people too”.

(P6:PI)

Improved patience was perceived as important for some participants’ personal development:

“I discovered that I was more patient than I thought I was. Eh, I could learn new skills if I just put my mind to it and thought about it.”

(P27:PI)

**Controlling anger:** Some participants described how their sense of responsibility towards the dogs impacted on their motivation to manage their anger more effectively:

“I have always been angry at things in life and found it hard to control my temper, part of the reason I ended up in prison. But I changed when I went on the course and worked with Missy, an 8-years old staffie cross bulldog that liked things quiet.”

(P20:WS)

Improvements in the management of emotions were related to their understanding of behavior generally, and this was perceived by some to be a worthwhile skill to obtain:

“I’ve changed… Anger and my attitude and that. I’ve got new skills.”

(P13:PI)

Being involved in the program also helped some individuals to manage feelings of anger at their situation and to cope within the prison environment:

“I was on the course when my dad died and found being on the inside hard. Having Paws for Progress to go to was good for me and helped me control my anger.”

(P33:WS)

**Negative experiences:** Around a quarter of participants (27%) described negative experiences. In the pilot phase, eight participants (out of twelve) raised concerns about sharing the workspace or with staff engagement. These concerns were subsequently addressed with a new workspace and dedicated prison officer to work on the program. Most of the remaining negative comments related to challenging aspects of participants’ experiences with the dogs, but almost half (44%) simultaneously described positive aspects of these experiences:

“It’s been good experiences. It’s hard at first, working out how to get a connection with a dog, but once you’ve got that it’s sorted.”

(P11:PI)

“Gutted. He got rehomed. Happy as well. I felt proud of myself.”

(P22:PI)

Other aspects that were potentially upsetting, such as learning about the individual dogs or their experiences, were also related to positive outcomes in terms of helping the dogs:

“I came down here, it shocked me when I was doing it. Just like wow. The first time you see them, the way they act. You feel upset when you see them. Then week by week you want to improve on the dogs so you keep your attendance going. Keep your attitude towards dogs. Speak clearly to the dogs. Giving it the right motivation. Then week by week that dog will just keep improving.”

(P57:PI)

## 4. Discussion

All program participants were positive when describing their experiences on the DTP and articulated beneficial outcomes for themselves and others. Thematic analysis highlighted a broad range of perceived benefits of a DTP that include positive psychological, social, and vocational outcomes [9]. Nine key themes were identified: Dogs, Positive Effects, Motivation, Charitable Purpose, Self-Efficacy, Improved Skills, Social Impact, Impulsivity, and Emotional Management. These themes mapped onto the program’s aims of improving behavior, increasing educational engagement, developing employability skills, and enhancing well-being (see Table 1). The perceived benefits are also congruent with those previously identified by participant and staff evaluations of prison based DTPs [10,13,15,20,22,27,28]. Although there are a variety of program models, AAIs encourage participants to interact positively with animals and people in a non-threatening and supportive environment [16]. Arluke [16] proposes that animal-assisted activities with at-risk youth primarily shape their social experiences, in terms of exposure to close relationships with animals and humans, softened hierarchies, new perspectives, easy successes, and manageable challenges. The inter-related themes identified are congruent with this interpretation, which also highlights the importance of evaluating the contribution that HAI makes towards outcomes, rather than trying to isolate this aspect from other program features [16].

In their recent review, Cooke and Farrington [9] categorized the reported benefits to DTP participants under several domains. Each of the themes identified in the current study can be aligned with the domains described: self-control (Impulsivity, Emotional Management), increased self-esteem and self-efficacy (Self-Efficacy, Motivation), increased empathy (Dogs, Charitable Purpose), improved social skills and prosocial bonding (Social Impact, Charitable Purpose, Dogs), increased emotional intelligence and emotional well-being (Positive Effects, Emotional Management), and increased employability (Improved Skills). The clear overlaps in how participants perceive their experiences indicate that, despite considerable variation in DTPs, similar processes are likely to underpin the positive outcomes described [16,24]. These domains can also be related conceptually to factors influencing future desistance from crime [9,22,28,51] or public health needs in this population [39,45,46].

The most prevalent theme related directly to participants’ experiences working with the dogs, including feelings, and attitudes towards dogs and training methods. Opportunities to engage in human–animal interaction in DTPs are central to the positive experiences reported by participants, in terms of enjoying interactions, bonding, providing emotional support, and facilitating social interaction [14,15,16,28]. A core feature of DTPs is the promotion of increased compassion and empathy for others, first given an outlet in working together to meet the dogs’ needs and then extended to other people, improving social relationships and prosocial behaviors [14,16,19,28]. Due to participants’ improved attitudes and behaviors towards dogs, and their enthusiasm for sharing their knowledge with family and friends, this theme also identifies potential benefits for dog welfare. A few participants also described their negative emotional experiences when a dog left the program, but most demonstrated their capacity to cope with this challenge by simultaneously identifying a positive outcome for the dogs or themselves [15,16,27]. The context of mutual rehabilitation had an impact on participants’ engagement and perceived outcomes, with many participants also identifying parallels between their own experiences and those of the dogs [19,20,28].

The theme of Positive Effects (enjoyment, change from institutional environment; therapeutic effects including bonding, and improvement in mood and wellbeing) is consistent with previous descriptions of normalizing effects, including reduced stress, enhanced mood, and a sense of connection to the outside world [15,27,28]. In terms of perceived therapeutic effects, participants often have limited experience of nurturing and supporting relationships [34,35,36]. AAIs provide opportunities to develop positive social relationships, as well as affectionate relationships with animals, and experience the emotional benefits of such connections [16]. The potential to have a positive impact on psychological well-being is an important aspect of DTP programs, given the significant mental health needs identified for young offenders (and others in custody) [45]. A lack of engagement in training and education opportunities is characteristic of this population; enjoyment is related to Motivation and Improved Skills, particularly in relation to fostering positive learning experiences that enhance educational outcomes [16,52]. Given the custodial context, participants’ enjoyment could potentially lead to negative perceptions of a program in the wider community. However, local communities generally view DTPs positively, because of the perceived capacity to rehabilitate inmates and dogs, and provide a useful service to society [7,12].

Enhanced Motivation (enthusiasm, rewarding experiences, and an enhanced sense of commitment and responsibility) is frequently identified as a beneficial outcome of DTPs [13,14,15,16]. Both the rewarding nature of participation and enthusiasm for the program were perceived to underpin participants’ engagement with the program’s activities and aims. Some participants articulated how the responsibility and affection they felt for the dogs, and the rewarding nature of these interactions, fostered a sense of commitment that helped them to rise to the challenges posed by animal training and care, or educational activities [16]. The high levels of Motivation reported by participants are validated by attendance, completion, and continuation rates, particularly given participants’ previously low levels of engagement with education and training (see Table S1); only four participants did not complete the program (all due to either transferal or release), cases of non-attendance due to choice were rare (attendance below 100% in <3 sessions per year) and 91% of participants progressed to an assistant or peer mentor role on program completion.

Most participants valued the opportunities afforded by the program to help or teach others; Charitable Purpose provided a positive prosocial focus in what can be an isolating and stressful environment [15,16,27,28]. A focus on helping others in DTPs is consistent with proposals for a shift towards a ‘strengths-based’ approach in criminal justice, focusing on the positive contribution an individual can make rather than on the perceived deficits of offenders [51]. McNeill and Maruna [51] recommend that the development, encouragement, and facilitation of opportunities to help others should be at the heart of effective practice with offenders. Some participants also described the positive effects of recognizing that they could enjoy engaging in prosocial activities, fostering the development of a prosocial identity. Similarly, Furst [28] described how adult male and female participants felt empowered by DTP programs, which enabled them to view themselves as prosocial and to engage in a worthwhile activity to benefit others, a process that was seen to facilitate desistance. Generativity, the desire to contribute positively and help others, is important because long term desistance frequently requires former offenders to redefine their personal identity as someone who is ‘making good’ [28,51]. Prosocial activities and commitments are seen to provide a sense of purpose and meaning to former offenders, and to legitimize the gradual transition toward a prosocial identity [51].

Many participants described an enhanced sense of Self-Efficacy (sense of achievement, confidence, sense of autonomy, problem-solving skills, aspirations) [9,15,22,27]. Self-Efficacy can be directly related to the asset-based approach that is common to many DTPs; staff focus on building upon the strengths of participants, practicing and developing new skills, and setting participants up for success [16]. This approach also clearly parallels the positive reinforcement training methods used with the dogs within the program [53,54]. Arluke [16] highlights that training dogs provides opportunities for participants to be trusted with responsibility and be a decision maker, enhancing a sense of autonomy and competence, and fostering an increased sense of self-worth [55]. The challenges faced within DTPs are manageable and participants are highly motivated to overcome these, by managing their emotions and demonstrating patience and self-awareness [16]. Improvements in problem-solving skills can be effective in reducing challenging behavior, improving communication, and repairing relationships in young people [56]. Like many other DTPs, this program was designed to encourage participants to progress towards new roles that signal successful advancement, and to celebrate achievements [16]. For some participants, enhanced Self-Efficacy was also associated with aspirations for themselves or for others who might benefit from the program.

DTPs often incorporate vocational or educational components that aim to enhance employability [9,10,14,15,21,22] because employment helps with the transition back into the community and contributes to desistance [22,32,33]. The human-animal context is important in fostering a calm and positive learning environment in which participants are simultaneously relaxed and engaged [16]. Most participants identified Improved Skills in relation to either employment or education. These positive perceptions are consistent with educational attainment on the program (at Access and Intermediate levels, Scottish Qualification Authority); all participants chose to complete optional qualifications, for many this was their first qualification, and almost all more than doubled the number of previous qualifications gained. Importantly, many participants highlighted their enjoyment of learning experiences [52], which facilitated an increased confidence in their abilities to engage in learning, develop skills, and pursue employment opportunities.

Social Impact describes the enhanced social connections facilitated by participants’ positive experiences working with others, the development of mutually supportive relationships with peers, improved communication skills, or improved family relationships. A few participants described a perceived impact of the program on their parenting style, indicating that DTPs may be a suitable approach for targeting parenting skills [27,28]. Many participants explicitly described the role of the dogs in providing a positive focus for their interactions with others, or in facilitating a sense of collective effort and responsibility. Enhanced social interaction, in terms of an increased willingness to participate in social exchanges [14,28] and improvements in the quality of social relationships, is considered a core feature of DTPs [9,13,16,22,27,28]. Dogs enhance social contact [57,58] and facilitate communication, effectively providing a ’communication bridge’ for positive social connections [5,19,59]. The promotion of prosocial and effective teamwork is a central component of DTPs, and the collaborative efforts of program participants and staff also fosters a sense of belonging to a community [16]. Enhanced social integration is associated with improved health and well-being, and the likelihood of future desistance from crime [45,59].

Self-reported improvement in institutional behavior and self-control were categorized under Impulsivity. Improved institutional behavior is commonly related to enhanced self-control in DTP participants [7,13,15,22]. Impulsivity is associated with increased aggression, and antisocial and offending behaviors [22]. However, previous findings are partially confounded by the strict criteria for participation in many DTPs, which require participants to maintain high standards of institutional behavior [9,15,22,27]. Although institutional behavior was not a criterion for program participation, several participants expressed concerns about exclusion and considered the potential consequences of their behavior, both for themselves and others.

Emotional Management includes improved patience and controlling anger, and is congruent with previous reported improvements in emotional intelligence and reduction in hostile emotions [14,15,22,27,28]. Some of the participants also described the development of coping skills to deal with challenges, both within the program and prison environment more broadly [14,15,28]. Developing an understanding of training methods, which focus on reinforcing positive behaviors and building on the dogs’ strengths, is considered to contribute to improvements in the management of emotion by DTP participants [16]. Taking the perspectives of others is also encouraged, improving empathy and emotional intelligence during interactions with dogs and other people [16]. Enhanced emotional intelligence has been reported to impact on behavior and relationships outside of the program, such as relations with other staff members, peers, and families [22,27,28].

Overall, all participants described positive experiences and perceived benefits, and each theme was referred to by most participants. The themes identified are broadly consistent with previous DTP evaluations that have used similar research methods. For example, Davis [14] also examined a community service model with male young offenders and identified five themes that could be aligned with themes reported in this study; developing a relationship (Dogs), increased sense of responsibility (Motivation), work skills of learning and teaching, and technical skills (Improved Skills), communication/social skills (Social Impact), and increased patience (related to aspects of Emotional Management and Impulsivity). Although there were no equivalent themes for Positive Effects, Charitable Purpose or Self-Efficacy, equivalent themes for each have been reported in at least one previous evaluation [15,27,28]. Currie’s [15] evaluation of a service animal program with adult male inmates identified six positive emotional outcomes and seven practical outcomes; despite the different structure, at least one of these outcomes could be aligned to the themes identified in the current study, with the exception of Social Impact. An additional theme describing a positive impact on the prison environment was reported [15], as previously identified by DTP participants [27,28] and prison administrators [22]. Although Davis [14] also did not report this as a benefit, it remains unclear whether this reflects a distinction between service animal and community service models, or another aspect of these particular programs or the evaluation process. It is also important to highlight that some themes were less common than others, suggesting that not all perceived effects will be experienced equally by participants. This variation also highlights the need to recognize the limitations of any one program in addressing the multiple needs identified in this population, as explained here by a peer mentor:

“But obviously it affects people in different ways... It’s not gonna turn everyone into a proper angel overnight... aye, that’s the way some folk’ll be, they’ll be under that impression. But it is going to improve patience, socialising skills, might seem like small things but they make a difference.”

(P4:PMI)

To allow others to assess the relevance and generalizability of these findings, the methodological strengths and limitations of this study are considered [25,26]. The research methodology is reported in sufficient detail, and participant and program characteristics are described to situate the sample. The relative prevalence of themes and grounding these in examples allows others to evaluate the interpretation offered here, and in relation to the specific context described [25,26]. For example, a lack of methodological detail can make it difficult to assess how the perceived outcomes described by participants have been shaped by the focus of the interviews conducted [11]. In this study, several topics were prompted during interviews and closely related themes would be expected to be more prevalent [22]. However, many aspects are less directly attributable to a specific question (see Tables S4 and S5). Unlike most DTPs, this program did not have strict selection criteria and the perceived outcomes are therefore not limited to those individuals most expected to succeed on a program [9,11,24]. However, participants were not randomly allocated to the program, and those who choose to participate in DTPs may differ from those who do not engage with such programs [9,11]. It was not feasible to conduct comparable qualitative evaluations for an appropriate control group; this is a key limitation of qualitative DTP evaluations which will need to be addressed despite the challenges of conducting research in a custodial context [7,11,24,47]. Nonetheless, the program appeared to be effective in engaging those with low levels of prior engagement with education or training opportunities, for example, as indicated by participant characteristics and program records.

The principal researcher was also primarily responsible for the development and delivery of the intervention under evaluation, with support provided by partner organizations, and as such was a principal stakeholder [11]. However, there were no DTPs established in the UK; this program was initially introduced as the core component of a research project and resources were limited. Combining the roles of practitioner and researcher had both benefits and costs, and reflects a conflict of interest that requires further reflection [25,26]. In terms of theoretical orientation, positive outcomes were anticipated based on previous experiences in researching HAI and delivering relevant services (e.g., Therapet visits). However, during program development, a review of the evidence available on the efficacy of DTPs highlighted methodological issues which constrained the evaluation of some of these previous findings [9,11]. In practical terms, the familiarity of the researcher to participants led to an excellent rapport, and high levels of voluntary participation with the evaluation process [9,15] although this may have also introduced a positive bias in participant responses. Moreover, the commitment to service development and delivery led to time constraints on the research process and these are reflected in several limitations. For example, peer debriefing was used to provide credibility checks but a formal process of response checking with participants, to assess the validity of researcher interpretations, would have been beneficial [15,25,26]. Similarly, gathering data from different groups, such as prison staff [13,15] would have allowed for triangulation with participants’ perspectives, and potentially identified other themes and issues [13,15,22]. Financial constraints precluded an external evaluation process, beyond institutional review processes [49], but this would be a desirable component of future evaluation. External evaluation would ideally be triangulated with internal monitoring to avoid the limitations of each method when used in isolation. For example, external evaluations of programs have generally been conducted over a short period and, due to the small numbers involved in these programs, sample sizes are small and relative prevalence of themes identified cannot be gauged [14,15,27,28].

The experiences of participants should be integral to DTP evaluations but there are limitations in relying on self-report methods, particularly if these are considered in isolation from other measures [9,11,22]. For example, most participants wanted to progress to a peer mentoring role and, particularly given the researcher’s dual role as program instructor, they may have felt obliged to be positive in their evaluations of the program. Similarly, some participants described a sense of ownership and collective responsibility in ensuring the program succeeded, which may have led to a reluctance to discuss any negative experiences. However, participants were encouraged to be as open and honest as possible during interviews, and attention was given to all negative comments, which served to reduce positive biases in interpretation. These negative examples indicate that participants were generally comfortable raising perceived problems or identifying the challenges they had experienced. The veracity of participant responses is further reinforced by several participants’ descriptions of prior negative experiences and attitudes towards dogs, which would be perceived as undesirable; similar comments were systematically excluded in at least one DTP evaluation [15]. Negative comments were also used to identify the potential challenges experienced within the program [15,22] and inform program development, for example, in relation to improving the facilities. In the overall program evaluation, quantitative measures were collected in parallel with these interviews as a means of addressing these potential biases in participant responses and researcher interpretation. These included self-report measures that may also be subject to such biases, but independent institutional and educational records were collated to provide objective measures of participant outcomes. Overall, while there are important limitations to qualitative evaluations when taken in isolation, participant perspectives are considered to be crucial to the evaluation of the program because these contributed to program development, may be able to detect benefits that are less easily measured quantitatively, and allow quantitative measures to be meaningfully interpreted within the context of participants’ experiences [11,24].

A focus on outcomes, including ongoing monitoring and evaluation, is central to program development and delivery using the “5 Step Approach” [32,33]. Within this framework, programs are responsive to issues raised through monitoring and evaluation; the connected responsibilities as researcher and practitioner facilitated a higher level of awareness of related issues than would commonly be expected in either role. For example, the extent of participants’ concern for the dogs was not fully anticipated, but early interviews highlighted the importance of managing the emotional attachments between handlers and dogs. Subsequently, the progress of trainers and dogs were more closely intertwined throughout the course; efforts were targeted toward helping the dogs be rehomed, and this was a celebrated achievement, which signaled progression to work with more challenging cases [16]. Similarly, Charitable Purpose is aligned with the program’s core aim of improving engagement in education (see Table 1) because it was evident that motivation to learn was dependent on educational activities being embedded in the context of helping the dogs (e.g., developing a portfolio to promote the dogs for rehoming). It is evident that the researcher’s experiences as a practitioner influenced expectations regarding outcomes, and these expectations shaped the research process, potentially biasing the research towards evidence that supported these expectations. However, several processes were in place to counteract potential bias, including impartial and non-leading interview questions, peer checking of thematic interpretations, and ensuring transparency in data collection and interpretation (detailing the interview schedule, indicating the relative frequency of themes, and grounding these in examples). It is relatively common for qualitative researchers to become immersed in applied research, to better understand participants’ perspectives and design the research appropriately [25,26]. However, there are important limitations in using these methods in isolation and such approaches are best implemented within a mixed-methods assessment of program outcomes [9,11,24].

This study describes how participants experience a DTP and is an important component of the overall program evaluation, which will triangulate these findings with quantitative measures to examine whether these perceptions translate into measurable improvements in outcomes for participants [7,9,11,24]. These findings should not be generalized to AAIs more broadly because the outcomes achieved relate to one type of program at a single institution, which was designed to meet the needs of a specific target group [7,11]. Despite overall congruence between the themes reported by similar qualitative studies, there are inconsistencies which may reflect variation between programs and selection criteria, the focus of an evaluation, or in the interpretation and structuring of often inter-related themes and constructs. For example, institutional constraints precluded the dogs living on site for this program; this impacts on the amount and nature of participants’ interactions with the dogs, experiences of institutional freedom and normalization, and the level of motivation and commitment required from participants. Similarly, participants did not identify previously reported negative experiences that primarily seem to relate to living with dogs, such as overwhelming responsibility, or the potential for conflict with other offenders [15,22]. In addition, the methodological weaknesses acknowledged in the current study largely reflect those previously identified in other DTP evaluations, and may result from similar constraints on research design, and limit the conclusions that may be drawn [9,11,22]. A more comprehensive and robust evidence base is required to examine program efficacy, including how program type and format impacts on experiences and outcomes, and whether some program types might be more effective in meeting the needs of specific groups than others [11,24].

## 5. Conclusions

Male young offenders perceived a range of positive experiences and outcomes resulting from their participation in a community service dog training program. The themes identified highlighted the potential for human-animal interactions to facilitate positive experiences, and also the perceived significance of a mutual rehabilitation context for many participants. The rich insights provided by qualitative approaches help to identify those features which may contribute to a program’s success. However, these perceptions should also be triangulated with outcome measures to examine the efficacy of a program and inform practice. Such evaluations are challenging within a custodial context, but important given the potential benefits of rehabilitation for participants and society.

## Figures and Tables

**Table 1:** Frequency of themes (in bold) and sub-themes in initial post-course interviews (N = 66).

Theme/Sub-Themes	N (%)	Theme/Sub-Themes	N (%)
**Dogs**	**66 (100%)**	**Self-Efficacy ^1,3^**	**58 (88%)**
Feelings about dogs	64 (97%)	Sense of achievement	46 (70%)
Attitudes towards dogs	63 (95%)	Confidence	37 (56%)
Attitudes to dog training	58 (88%)	Sense of autonomy	25 (38%)
Parallels with dogs	32 (48%)	Problem-solving	20 (30%)
**Positive Effects ^1^**	**65 (98%**)	Aspirations	20 (30%)
Enjoyment	65 (98%)	**Improved Skills**	**57 (86%)**
Change from prison environment	60 (91%)	Employment ^3^ Education/Learning ^2^	42 (64%) 40 (61%)
Therapeutic effects	34 (52%)	**Social Impact**	**55 (83%)**
Improved mood and wellbeing	20 (30%)	Working together ^3^	53 (80%)
**Motivation ^1,2,3^**	**65 (98%)**	Peer support ^1^	34 (52%)
Enthusiasm	60 (91%)	Communication skills	22 (33%)
Rewarding	52 (78%)	Families/parenting	10 (15%)
Commitment and responsibility	37 (56%)	**Impulsivity ^4^**	**38 (58%)**
**Charitable Purpose ^1,2^**	**57 (86%)**	Institutional behavior	30 (45%)
Helping others	46 (70%)	Self-control	24 (36%)
Teaching others	43 (65%)	**Emotional Management ^3,4^**	**37 (56%)**
		Patience	28 (42%)
		Controlling anger	26 (39%)

Alignment of themes with program aims: ^1^ **Enhance well-being**: motivation, self-efficacy and positive prosocial focus. ^2^ **Increase educational engagement**: attitudes to learning, progress and achievements. ^3^ **Develop employability skills**: social competencies, emotional management, independence and team work, responsibility and decision making, problem-solving, working towards targets and goals. ^4^ **Improve behavior:** in the institutional environment and in the long term.

## Supplementary Materials

The following are available online at www.mdpi.com/1660-4601/14/8/945/s1, Table S1: Participant learning histories (accessed via Scotland’s National Pupil Database; N = 70). Table S2: Participant histories recorded on the prison system database (not mutually exclusive; N = 70). Table S3: Participant convictions, sentences, and offense categories recorded on the prison system database (N = 70). Table S4: Interview Schedule. Table S5: Summary of responses according to interview question (N = 66). Table S6: Frequency of themes (highest to lowest) and sub-themes identified in all coded sources (N = 77). Table S7: Additional examples of quotations relating to the themes and sub-themes identified.
