# Supplementary material for: “You Think You’re Helping Them, But They’re Helping You Too”: Experiences of Scottish Male Young Offenders Participating in a Dog Training Program
Source: Int J Environ Res Public Health. 2017 Aug 22;14(8):945. doi: 10.3390/ijerph14080945 (PMC5580647; doi:10.3390/ijerph14080945)
Supplement: Supplementary file 1 [file ijerph-14-00945-s001.pdf]

Supplementary Materials

Table S1: Participant learning histories (accessed via Scotland's National Pupil Database; N = 70)

**School Leaving Age (yrs)**	**N (%)**	**Attended further education / training**	**N (%)**
16+	20 (29%)	Yes	12 (17%)
15	1 (1%)	No	58 (83%)
14-15	31 (44%)		
<14	13 (19%)	**Number of previous qualifications**	
N/A (serving custodial sentence)	5 (7%)	0	33 (47%)
		<5	24 (34%)
**Type**		6-10	10 (14%)
Residential	6 (8%)	11-15	1 (1%)
Secure	11 (16%)	Unspecified qualifications	2 (3%)
Mainstream	39 (56%)		
Alternative	11 (16%)	**Highest level of qualification gained**	
No record	3 (4%)	None	33 (47%)
		Access	1 (1%)
**Recorded Difficulties at School**		Intermediate	8 (11%)
Unknown	3 (4%)	Standard grade	28 (40%)
Yes	59 (84%)		
No	8 (11%)		

Table S2: Participant histories recorded on the prison system database (not mutually exclusive; N = 70)

**Mental Health**	**N (%)**	**Adversity**	**N (%)**
History of mental health issues	28 (40%)	In care as a child	24 (43%)
Accessing mental health support in YOI	18 (26%)	Trauma as a child	38 (54%)
Suicide-risk management strategy	11 (16%)	Childhood neglect or abuse	27 (39%)
History of alcohol abuse	62 (89%)	Significant bereavement(s)	19 (27%)
History of drug abuse	52 (74%)	Multiple adversities	43 (61%)
History of substance abuse	65 (93%)		
Accessing substance abuse support in YOI	45 (64%)	**Offending behaviour**	
		Previous convictions	53 (76%)
		Previous custodial sentences	32 (46%)
		Offending behaviour (<16yrs)	39 (56%)

Table S3: Participant convictions, custodial sentences, and index offense categories recorded on the prison system database (N = 70)

**Convictions and Custodial Sentences**		**Mean (min-max)**
	Time in prison (months) before beginning programme	7 (0-33)
	Sentence Length (months)	39 (7-192)
	Number of Index Offences	4 (1-19)
	Number of Previous Custodial Sentences	1 (0-5)
	Previous custodial sentences: Time served (months)	8 (0-77)
	Total time in prison (previous and current sentences)	15 (0-83)
	Number Previous Convictions	5 (0-29)
**Index Offence Categories**		**N (%)**
Threatening	Threatening/abusive behaviour, offensive weapon, racial abuse	1 (1%)
Violence	Assault, assault to injury, police assault/resist arrest	30 (43%)
Severe violence	Assault to severe injury, permanent disfigurement, permanent impairment	18 (26%)
Life endangerment	Attempted murder, culpable homicide, murder	11 (16%)
Dishonest	Theft, shoplifting, theft by housebreaking, conspiracy, theft of vehicle, reset	3 (4%)
Drugs	All drug related offences	2 (3%)
Traffic	Traffic related offences	1 (1%)
Antisocial	Vandalism, breach of peace, malicious damage / mischief, reckless conduct	0
Breach	Bail breach, breach of community order, failure to attend hearing/court, breach of curfew	0
Recall	Recall to custody following license breach to serve extended / remaining sentence	1 (1%)
Multiple non-violent	Multiple non-violent offenses with index offence unclear (i.e. >3 offenses in multiple categories)	3 (4%)

Table S4: Interview Schedule[1]

1. Could you tell me about your experiences in the dog training course?	a. Did you enjoy it?<br>b. Did you find it useful?<br>c. Did you notice any changes in yourself as a result of taking part?<br>d. Did you like working in a group?<br>e. How did it compare to other programs/ courses you have done at the prison?
2. Could you tell me about your experiences with the dogs?	a. What part of working with the dogs did you enjoy most?<br>b. What did you think of the dog you worked with most?<br>c. Do you think it will affect how you are / what you will do with dogs in the future?
3. Was the course what you expected?	
4. Do you have any suggestions of what we could do to improve the course?	

[1]Prompts used as needed: You said that …..Could you tell me more about that / what did you mean by that? What was it like? How did you find that?

Table S5: Summary of responses according to interview question (N = 66)

Topic	N (%)	Topic	N (%)
**Enjoyment of program**		**Working with dogs**	
Positive experience	66 (100%)	Positive experience	66 (100%)
		Changed attitudes and behaviour to dogs	66 (100%)
**Usefulness of program**			
Not useful	0	**Comparison to alternatives available**	
Useful (unspecified)	10 (15.2%)	No experience of alternatives	8 (12.1%)
Useful single domain[1]	25 (37.9%)	As good as alternatives	3 (4.5%)
Useful multiple domains[1]	31 (47.0%)	Better than alternatives	55 (83.3%)
**Resulting change(s)**		**Expectations**	
No change	1 (1.5%)	No prior expectations	5 (7.6%)
Change (unspecified)	2 (3.0%)	As good as expected	12 (18.2%)
Change single domain[2]	8 (12.1%)	Better than expected	49 (74.2%)
Change multiple domains[2]	55 (83.3%)		
		**Improvements**	
**Working in a group**		No suggestions	6 (9.1%)
Positive experience	66 (100%)	Improvements not needed	38 (57.6%)
		Improvements suggested (activities, facilities, expansion)	22 (33.3%)

[1] **Domains:** Improved understanding of dogs (N = 39); learning (N = 30); ability to teach/share learning (N = 22); personal development (N = 21); future employment (N = 11); improved behaviour (N = 11).

[2] **Domains:** Management of emotions (N = 37); improved confidence (N = 26); social skills (N = 25); increased empathy and improved understanding of dogs (N = 24); learning and employability (N = 19); self-control and behaviour (N = 18); well-being (N = 16).

Table S6: Frequency of themes and sub-themes (and negative experiences) in initial post-course interviews (N = 66) and as a total of all coded sources (N = 77)

Theme/sub-themes	Interview	All	Theme/sub-themes	Interview	All
**Dogs**	**66 (100%)**	**77 (99%)**	**Self-Efficacy[1,3]**	**58 (88%)**	**67 (86%)**
Feelings about dogs	64 (97%)	74 (95%)	Sense of achievement	46 (70%)	54 (69%)
Attitudes towards dogs	63 (95%)	71 (91%)	Confidence	37 (56%)	41 (53%)
Attitudes to dog training	58 (88%)	64 (82%)	Sense of autonomy	25 (38%)	30 (38%)
Parallels with dogs	32 (48%)	36 (46%)	Problem-solving	20 (30%)	23 (29%)
			Aspirations	20 (30%)	25 (32%)
**Positive Effects[1]**	**65 (98%)**	**76 (97%)**			
Enjoyment	65 (98%)	76 (97%)	**Improved Skills**	**57 (86%)**	**66 (85%)**
Change from prison environment	60 (91%)	67 (86%)	Employment[3]	42 (64%)	50 (64%)
Therapeutic effects	34 (52%)	40 (51%)	Education/Learning[2]	40 (61%)	47 (60%)
Improved mood & wellbeing	20 (30%)	28 (36%)			
			**Social Impact**	**55 (83%)**	**65 (83%)**
**Motivation[1,2,3,]**	**65 (98%)**	**76 (97%)**	Working together[3]	53 (80%)	63 (81%)
Enthusiasm	60 (91%)	70 (90%)	Peer support[1]	34 (52%)	43 (55%)
Rewarding	52 (78%)	61 (78%)	Communication skills	22 (33%)	26 (33%)
Commitment & responsibility	37 (56%)	44 (56%)	Families/parenting	10 (15%)	12 (15%)
**Charitable Purpose[1,2]**	**57 (86%)**	**68 (87%)**	**Impulsivity[4]**	**38 (58%)**	**48 (62%)**
Helping others	46 (70%)	55 (71%)	Institutional behaviour	30 (45%)	37 (47%)
Teaching others	43 (65%)	52 (67%)	Self-control	24 (36%)	31 (40%)
			**Emotional Managemen[3,4]**	**37 (56%)**	**46 (59%)**
			Patience	28 (42%)	34 (44%)
			Controlling anger	26 (39%)	34 (44%)
			**(Negative experiences)**	**18 (27%)**	**18 (23%)**

Alignment with program aims:

[1] **Enhance well-being**: motivation, self-efficacy and positive prosocial focus.

[2] **Increase educational engagement**: attitudes to learning, progress and achievements.

[3] **Develop employability skills**: social competencies, emotional management, independence and team work, responsibility and decision making, problem-solving, working towards targets and goals.

[4] **Improve behavior**: in the institutional environment and in the long term

Table S7: Additional examples of comments relating to themes (in capitals) and sub-themes identified.

THEMES / sub-themes	Quotations from interview transcripts (participant number and source type)
**DOGS**	
**Feelings about dogs; Attitudes towards dogs; Attitudes to dog training; Parallel experiences with dogs.**	*"I enjoyed working with the dogs. I enjoyed teaching them how to do tricks and obviously learning how to be patient with dogs and just taking your time. The clicker training and all stuff like that. I just enjoyed all of it."(P18:PI)*
	"Yeah, I have really enjoyed it. It's been a good experience because it's something I've never actually done before. It's like actually working, working with dogs. Working with dogs and that. I've fair enjoyed it."(P2:PI)
	"Definitely. I'd been brought up with dogs but I never really thought about behaviour, feelings, stuff like that. So basically when I get out I'll just show some of the qualities I've learned in here and just try and put it into my own dogs. I think I'm going to bond a lot more with dogs now I know how to train them and stuff like that."(P60:PI).
	"I think the best bit was probably when she started running. Just seeing that wee face going. That was hilarious. Thought it was funny just to sit in a certain bit with my trainer, but when I started telling everybody that's what she did, she sat next to me instead when I was training her. That dog had a sense of humour! All seriousness, at first she was dead reluctant and after a while she was dragging me along with enthusiasm instead of me trying to motivate her. Brilliant dog. Couldn't think of a better dog to be honest. See just cos it's the first one. Don't think it'd be the same again. See we click, it's funny but we do."(P51:PI)
	"Well ah think Paws for Progress is good because it's different. It also gives you a chance to learn new things. I think the best thing about this programme is that you get to train a dog to do what it's told like sit, paws or tricks! Paws for Progress should be available in more jails and in more communities."(P51:WS)
	"I enjoyed teaching the dog new things and the dog teaching me new things."(P20:PI)
**POSITIVE EFFECTS**	
**Enjoyment**	
Novel and engaging; Interactions with dogs; Varied activities; Social dynamics/team work; Positive learning experience	*"It's something different. I like this, know what I mean? It's something different. This is different, see cos I've never done this outside or nothing, it's different so I like it. I like this cos it's something I've never done before. I like working with dogs and that. It's good."(P38:PI)*
	"I've done a full course now. I enjoyed the people from the zoo, the vets and the people from the SSPCA coming in. I enjoyed it all."(P59:PI)
	"I dunno, I thought we were just coming down, taking the dog out but no that we were doing various stuff. We're not just out with the dog every day, we're watching videos or other folk having dogs speaking to us. Better than what I thought anyway, more enjoyable."(P56:PI)
	"Aye I really enjoyed it. I enjoyed working in a group. I think it brought me out myself more."(P63:PI)
	"Brilliant. Now I know all them down here as well. Some laugh down here. It's not as if it's like, strict or shite or that. You get on with them, they get on with you and we all do what you tell us. It's alright, it's enjoyable."(P54:PI)
	"I thoroughly enjoyed it. And I found it very useful, in the way that I've been able to better myself, and will be able to use the learning, and things we've been working on, on the outside too." (P9:PI)

**Change from prison environment:** Comparison to other activities; Normalising effects.	*"It's a different thing seeing the dogs when you're in the jail cos it gives you a wee sense of being outside if you know what I mean. It's really good, I enjoyed it so much."(P44:PI)* <br>*"I haven't done a lot of other programmes or courses while I've been here, but I find this to be the best thing that I've done in here. It's different, and it's very useful."(P26:PI)* <br>*"This is probably the best course I've done. I'm not just saying that, it is. Everything else, see if you go to a work party or if you go to education you're doing the same thing every week but in here sometimes you're doing similar things but other times you're getting a dog every week and getting the dog to do something new."(P14:PI)* <br>*"Aye. Cos it's working with all the dogs and all that and going into an environment I've no been in so. Obviously cos I'm in prison I'm used to it, it's my environment. But when people come in they are basically coming into our house so we don't really bother. But going outside and going into a new environment, I thought that would be a bit weird cos obviously you've not got people telling you all the time - you can't go certain places, you can't do this, you can't do that. You've not got an officer walking about with you telling you what to do when you're trying to train the dogs so it's a bit different."(P20:PI)*
**Perceived therapeutic effects** <br>Calming and relaxing; Building trust/ bonding with the dogs; Mutually beneficial interactions	*"I've done programmes but this was probably the best, and it's a work party. Different. Bit like a programme/work party isn't it? Good. More relaxed. Obviously you've still got the same rules and that but it doesn't feel like they're thrown right in your face…. It's good. The fact you get to go out and walk about as well, it's weird. This you just relax. You learn something."(P51:PI)* <br>*"Yeah you get to relax and you get to spend time with the dog. Get bits of one to one. Sometimes it's useful stuff in the other programmes, but there's maybe a group of 7/8 in the class and a tutor so you don't really get a bit of one to one. Makes it good in here that you can get a bit of time to yourself."(P57:PI)* <br>*"I thought, I'm not really good with animals but I seem to have took to that no bad. Just like, teaching how to sit, stand, paw, taking her out on walks. Running with my dog. Cuddles and that. You don't think you're going to run about with the dog and give it cuddles and that. You sit down with the dog and the next minute the dog is jumping all over you. It's a laugh but, it makes you feel good."(P51:PI)* <br>*"I very much did enjoy the course… as soon as I came down here it kind of made me want to get out my bed and stuff like that cos I enjoyed it. Kind of gave me a good time just to relax and socialise. Having the dog there is very therapeutic. I've enjoyed it very much."(P60:PI)* <br>*"I liked Shane, I did like Shane the best, I think it was cos I got a real bond with him. It was - we just clicked, I suppose. And he really liked me I think. And I liked him. And he think he felt it. Think he felt the brotherly love!"(P7:PI)* <br>*"The dog became more confident with me. When I first got him, he was unsure a bit. You just need to keep learning how to do things and that. Letting him know he's alright. He can trust you."(P67:PI)* <br>*"What do I like best? That's a hard one. I think just sitting with her, when you've got time after the walk and the training, getting to know what she's like, it's that bit of bonding time with her. Just sitting the two of you, and she'll just sit there looking to you, you know what I mean?"(P12:PI)* <br>*"Mojo was, I don't know, he was eh, maybe he's not as cool as other dogs or as cool looking, and things like that but I don't know man. It sounds cringe worthy but it's like the connection, know what I mean? He's a brilliant wee dog. I don't know, I wouldn't have changed him for any other dog for like, anything, know what I mean?"(P40:PMI)*

Improved mood and wellbeing

Positive mood; Engaging in positive activities

"I think Paws for Progress is a positive thing that's interesting and good for Polmont. Paws for Progress should be available in different jails because it's like a bit of the outside that gives you more positive thoughts!"(P50:PI)

"Aye it's good. Because you're up early, working hard and all that, you're tired and relaxed at night. You feel better."(P70:PI)

"I feel a lot better, to be doing something more constructive….So this is proper, making good use of the time. And it's good knowing you're coming down to it, it's something to look forward to."(P3:PI)

MOTIVATION

Enthusiasm

Sense of enjoyment; Programme delivery; Sense of gratitude

"I think it's spot on to be honest. Perfect the way it is the now. Just keep everything as it is, aye."(P10:PMI)

"Keep up what you're doing that's the best thing. Aye, it's good. It's an excellent thing to do in a jail."(P17:PI)

"I think Paws for Progress is a great project to be a part of. It gives prisoners good opportunities to get qualifications and certificates. It also gives the dogs that take part a better chance of getting rehomed. It's a work party that I personally enjoy coming to, I think this project should be an on-going thing!" (P54:WS)

"Working with Paws for Progress was a privilege and I am very grateful for the opportunity. I would highly recommend Paws for Progress to anyone who has a passion for helping animals and / or would like that second chance to change for the better." (P8:WS)

"Working with Paws for Progress was a privilege and I am very grateful for the opportunity. I would highly recommend Paws for Progress to anyone who has a passion for helping animals and / or would like that second chance to change for the better."(P8:WS)

"Just my way of saying thanks for all your help when I was on the course, you give so much help to people and expect nothing in return, truly amazing. If I can do anything at all in the future let me know. More than happy to help, the course helped me a lot so I kinda owe yous. And the very least I can do is stay in touch, your course really helped me. Thanks again."(P34:WS)

Rewarding

Positive training experiences; Getting something back; Personal development gains

"See like when you get them to do something after spending so much time with them. Getting Marty to sit down and give you a paw and that. It's good, like when he first come on the course he done nothing. I enjoy that. Seeing an improvement in the dog. That's probably the best thing about it."(P14:PI)

"The main dog I was working with was Blue and I loved that dog. The bonding between the both of us. The dog wanted to take his time and find different situations. It was a challenge. So yeah, it's good too. You're in the jail, you never think you're going to see a dog but come here, you can make a real difference to a dog."(P57:PI)

"Training them. I enjoyed training them the most. Being able to say, I taught this dog to sit, that was me that done that. That was what I liked best."(P7:PI)

"Mojo's a brilliant wee dog. Easy to get on with, if you put enough effort in then he gives you it back so. You can't expect someone to go in and just start man handling. You just go in and show him a mutual understanding then it's all good."(P40:PI)

"This course has helped me realise how smart dogs are and how much progress it has – on you and the dogs. It helps me and I will now know how to treat and train dogs when I am lib'd from Polmont. It's been a fantastic course and it helps me to be patient and improve my skills."(P57:WS)

**Commitment and responsibility**<br><br>Sense of challenge; Compared to alternative activities; Importance of others sharing responsibility; Sense of shared ownership and sharing success	*"Aye, it was better than I expected. I didn't think you would be training them that much. It's a lot – it's full on. Responsible for everything the dog does. Learned a lot about it as well."(P52:PI)*<br><br>*"I think there was more to the course than I expected. Like when you explain it from the start it seems quite basic and all that. But like, you think you'll be able to get it and all that dead easy. But I think there was loads of wee difficult things in it. There was more to it, know what I mean? Challenging."(P32:PI)*<br><br>*"I think Paws for Progress is the best. The staff are very good. It has helped me stay calm and stay out of trouble. It is the first work I have been fully committed to."(P62:WS)*<br><br>*"I've not finished anything I've done before. I start it, then I just ditch it. It doesn't interest me. I only do things that interest me. This is different. It's good."(P38:PI)*<br><br>*"Aye. In fact see to be honest with you, I never expected anything. I expected to come up here, sit for a wee few weeks then patch it, go back to gym training again. But ended up sticking, it was good so…"(P20:PI)*<br><br>*"It was good, it was really really good. And I liked Shane, it was good that he was my first dog. He'll listen, he's well mannered. I like that, I really liked Shane. My second dog, now he was a bit of a handful. It was harder, although I still tried, I tried my hardest with him. But I really enjoyed it. And I'd do it all again, easily, happily."(P7:PI)*<br><br>*"But see, the way it's going the now, I think it's fine the way it is now, it's brilliant. You're going about it the right way, step by step, cos a lot of folk need that. Working it like this, a new course every couple of months, it's there. If people really want to do it, they'll do it. And if people want to get the gains from doing it, it's there for them and they can do it."(P4:PI)*<br><br>*"You need to be able to like, really want to do it. Not just coming on the course for the sake of it because you want to get out yourself or whatever. Because I think, I think you should really want to do it and put a lot of effort into it." (P24:PI)*<br><br>*"It's good because it's hands on, you get to be with a dog. You get a good laugh with all the boys but they take it seriously at the same time, it's not just a big laugh and a joke. It's something that everybody wants to do, everybody looks forward to a Wednesday and a Friday when they're coming down and getting a dog, it's good. It's different but it's brilliant." (P27:PI)*<br><br>*"As a peer mentor I was given more responsibilities, at the start of each session I would set the room out in preparation for the dogs arriving, and then tidy up and put everything away at the end. I would do my best to help the new students with their dogs and with any written work they were struggling with. I would also assist our teacher throughout the morning and help to ensure our sessions ran as smoothly as possible."(P8:WS)*
**CHARITABLE PURPOSE**	
**Helping others**<br><br>Helping dogs by improving welfare and rehoming; Mutually beneficial interactions	*"It would be just as rewarding working with the dogs as it would with the other boys as well. It's a reward - seeing boys getting jobs out of this, it's just as much of a reward as seeing dogs get rehomed, you know what I mean? It would be good to have guys who've been through this, who have done well out of this, having a chance to be a role model for others coming through as well."(P4:PMI)*<br><br>*"I would say probably the best bit was completing my Good Companion Award with my dog. It also means that he's a good dog and it's a step forward to everyone seeing that the dog is a loyal, good companion."(P53:PI)*<br><br>*"Aye, definitely. And it helps the dogs get rehomed. Like see Ollie, it was clear, it helped him get rehomed faster than it would have. And see cos they get trained up, there's less chance of*

them getting brought back too, cos they'll be better behaved and that too. It's good then, isn't it?"(P3:PI)

"We've been looking after the dogs, training them. Obviously to stay, wait. Basically manners you need for a dog to be safe outside. Wait so they don't get knocked down. Handling for first aid work. Teaching them basically to behave well to help them get rehomed. Maybe started with an unsettled life and we rehabilitate them. Aye I loved it. Every bit of it."(P56:PI)

"I think Paws for Progress should continue because it rehabilitates the dogs and helps rehome them. We are also learning to be patient and calm. It is also good to know that we are making a difference to the dogs' lives."(P59:WS)

"I was helping with Mojo as a stooge dog for Harley and Diesel, I worked Mojo as a stooge dog for them, to try and help them to be more calm around dogs. I think he's relatively good around other dogs. They done a bit of agility and that with Mojo nearby. Stuff like that really helped them." (P40:PMI)

Teaching others

Training the dogs; Effective teaching also involves learning

"I enjoyed everything really. Training them and watching the dog getting better. Watching dogs coming in and they're not good at things. They're not really learned but we learn the dogs good in here. We've taught them a lot, manners, to walk nice on the lead, to take food nicely and stuff like that."(P24:PI)

"Well, one the things I did in the dog training course was teach a dog to have more confidence in itself... to maybe feel better."(P6:PI)

"Just noticing the difference you've made from the first day you meet the dog until when the dog leaves. Just the gradual steps involved. You start to notice them. You feel good about it as well. You feel happy with yourself. That you're teaching an animal how to do the basics."(P33:PI)

"I enjoyed teaching the dog new things and the dog teaching me new things."(P20:PI)

"Cos if I get a new dog when I'm outside, it helps you teach them. You can train it from when it's just a pup and all that. You learn things, what not to do to them and what to do. Like before I'd usually shout at a dog if it done something wrong. I'd shout at it probably. You don't do that cos it can make it scared of you. There's better ways of teaching." (P30:PI)

SELF-EFFICACY

Sense of achievement

"Kia, aye a cracking wee dog. She was quite scared once she came in. I watched her trust us a lot more. Because like, obviously I've been good to her and that. I rewarded her when she had done good stuff and she learned. She learned more about stuff and then learned about manners, walking good on the lead. She's a lot better now and she's been rehomed. Felt good. I felt proud of myself. Proud of her as well for being fast at learning all the stuff she did."(P24:PI)

"You get a lot of useful information and skills that could also help on the outside, such as patience, teamwork and the ability to read a dog's body language. I have learned a lot whilst attending this course and I am proud of what I achieved."(P54:WS)

The training. Knowing that he sat nicely when people came to meet him at the kennels made me feel good. Before he used to jump up on people and all that. Obviously people he's just met. But I trained him, he learnt manners. Aye that was my achievement but that wasn't just my achievement. My achievement was that I helped him getting rehomed, that was my achievement."(P20:PI)

"Seeing a difference in the dog from the start to the end just knowing it was you that made a difference. You kind of feel good for yourself cos you feel like you've really achieved something."(P60:PI)

"Seeing the difference in the dog, from the start and then later on, it's good, it's a good feeling. Knowing you taught them, you did that. It's brilliant, to get that sense of accomplishment, it's really good."(P11:PI)

Confidence

In social interactions; In own abilities

"Aye, you just meet people for like the first time you're like, not too confident in yourself and that. Then you get to know them, and you can like talk to them. Aye, coming out your shell a wee bit, that kind of thing."(P17:PI)

"I've noticed a few changes. Just noticed that I can do things that I put my head to. At first I thought I wouldn't really have made a difference, but I feel that I have made a bit of a difference. I got to work with a few of the boys and that so I made a good couple of pals too. Just, really liked the course in general. Really good." (P33:PI)

Sense of autonomy

Thinking independently

"This is more free, more freedom and all that. It's alright. It's not something you have to do, it's something you want to do."(P15:PI)

"It's thinking outside the box. Most of it at the prison is to do with inanimate objects and working with walls and wood and that. This is working with live things. It's better, builds your life experience."(P28:PI)

"It's like the peer mentors. Give them a chance, see what they can do, stand on their own two feet and show that they can be trusted, that they can do that kind of stuff. It's good to give someone that kind of responsibility as well." (P3:PI)

Problem-solving

Setting goals and targets, monitoring progress

"Working with Diesel. Just putting him through agility and all that, to learn to work together. Trying to get him used to being around other dogs and to be alright round people. Help him know that it's safe. That's good isn't it? Brilliant."(P38:PI)

"Getting to see Harvey, build up a relationship with him, working with him and playing with him and all that. See at first, he didn't know how to listen at all, but it's all about getting to know him better."(P8:PI)

Aspirations

For self; For others

"I'm more… considering my future and looking forward to things and that, sounds good."(P15:PI)

"I think it made me grow up a bit and look to the future and see what I want to do."(P18:PI)

"I think that Paws for Progress has been good for me and Polmont and all the lads who have attended. I think it's important that it continues onto the future."(P38:WS)

"Aye, it's good cos I'll keep out the jail and I'll get to work with dogs. I've always wanted to work with dogs."(P20:PI)

"Try to get this - you know, after the two years starting out here, you can really do something with this. You know, this would be great for lifers, really really good. Like guys from the open as well, they could be doing this to their and others advantage. It could be good for more than just the rescue dogs, think about stuff like the Guide Dogs as well. So hopefully you'll be able to set something up like that too. See how it goes, eh?"(P4:PMI)

"As well as seeing Paws for Progress installed as a permanent full-time programme at Polmont it would be great to see it expanded to other prisons across Scotland…. For example, a long term prison would be a good place to set up a programme for training assistance or guide dogs as this takes a lot of time to do and the guys there have a lot of time to do it in. In a short term prison you could set up something similar to Polmont where the prisoners train rescue dogs for rehoming. Again, these programmes would be something that would benefit everyone involved. The number of dogs being successfully rehomed would increase, freeing up space in rehoming centres. More jobs would be created, it would increase education and learning within prisons, and it could help reduce reoffending. It would also be a huge help to the charities who train

assistance and guide dogs and to the people that require them, as more dogs can be trained quicker and cheaper."(P8:WS)

"As well as expanding Paws for Progress across other prisons I think it would be a brilliant idea to target young people before they go to prison by setting up dog training programmes in schools or youth centres. Personally, I honestly believe that if I had known about Paws for Progress before I came to prison, I would not be here now."(P8:WS)

"I think it would be a good idea for this program to go to other jails and secure units, and maybe if I was given the chance to do a course like this I may have never ended up taking the path that I did and ended up in prison."(P38:WS)

"It now has its own room within the prison, it has a prison officer dedicated to helping with the running of it every day and has all the required facilities. So much hard work has gone into making Paws for Progress the success that it is, it would be a huge shame to see it all come to an end." (P8:WS)

IMPROVED SKILLS

Employment

Gaining new skills and confidence; Sense of challenge; Work experience and opportunities

"Paws for Progress is fun as well as educational and the best part is that it doesn't stop when you get out of jail. It carries on outside with placements and job opportunities."(P28:WS)

"Well I think it's going to get us a job when I'm out isn't it? That's good. Something good to do when I'm out."(P15:PI)

"Not only does it give you something constructive to do with your time in prison but you can also gain qualifications in subjects such as communications, numeracy, personal development and ICT. As well as all of this Paws for Progress provides work placements for almost all its students on release, some are even lucky enough to be offered jobs."(P8:WS)

"It's an area of work I'm determined to get into when I get out. I'll make the most of the opportunities you've made available through the course. I know what it feels like now to have a job you look forward to going to in the morning – it's a very different feeling."(P4:PI)

Education and learning

Enjoyment and engagement; Positive environment; Sense of achievement; Positive attitude to learning

"Not only are you learning new things you are having a good time while you're doing it so. I think that's maybe one of the biggest points. It's having that fun time, cos if you're having a good fun time then you have a good time doing it so you learn. I would recommend it for anybody else."(P60:PI)

"I think this is the only work party where you're actually learning about stuff. Every other work party I've been to, same stuff every day. Here you're doing different things every day."(P56:PI)

"I think one of the reasons the course was so good was because the staff were very kind and helpful, and encouraged everyone on the course."(P64:WS)

"The paperwork sometimes, I find that quite hard. But it's good. I've never wrote as much in my full life. This is the only time I've done writing. I don't do writing anywhere else. Down here's helped me with writing. See how we do so much writing? I'm always asking people how to spell things, they're telling me. I've registered, it's came in my head and now my spelling is better. That's good as well. I write more too. It's helped my writing and that as well down here."(P38:PI)

"I found it very useful, in the way that I've been able to better myself, and will be able use the learning, and things we've been working on, on the outside too."(P9:PI)

"Aye I learn lot better man. My spelling and my writing and all that has got a lot better as well cos I can think in my head and all that." (P12:PI)

"I enjoyed the written work we were asked to complete as it really helped me to improve my reading, writing and spelling. During my time on the course I received a lot of positive feedback about my written work as well as the training I was doing with my dog. This really helped my confidence which is something I was certainly lacking before. When I think about it, Paws for Progress did so much for me in such a short time. Not only did it help me change my behaviour for the better, it also helped me to improve my skills."(P4:WS)

SOCIAL IMPACT

Working together

Benefits for reaching common goals; Interpersonal skills

"It's actually better than I expected. My attitude with everyone else that was down here, it changed once the dogs were in. You got to see the proper side of them, working like that, and it makes it easier to get on with everyone. At the start, everyone might muck around while they're waiting for the dogs to come in, but everyone knows how important it is not to be like that with the dogs there, you can't be stressing them out so... Then you get to see the proper side of them, taking it seriously. Brings out the best in folk."(P5:PI)

"I thought it would've been hard, cos to begin with, the dogs don't know each other and they might not get on… But it's actually alright, they get used to being around each other quickly don't they, it's really good, better than I thought. And it's better when you get on with people in the group, we all work together to make it work, and that makes it 10 times easier."(P3:PI)

"Yes, I found I'm a lot more calmer and relaxed. Better working in a group and not just thinking about myself. Got to think about others too."(P9:PI)

"It's been an enjoyable course. It's been pleasant. It's made me build up better relationships, with the mentors in the class and then friendships with other people."(P53:PI)

"It's different. Decent group. Usually you get a group where you've got a few good people then you've got idiots or people you don't like. This one, everybody seems to get on with it and get a laugh and nobody bothers anybody. Just get on with it and cheer each other up. Keep the dog a safe distance and all that. Get on with it. I was surprised. I wasn't expecting it to be like, just come down and be like that - but I came down here and everybody's alright, makes it easier."(P51:PI)

Peer support

Peer mentoring; Sharing skills with peers; Understanding and empathy

"I very much enjoyed it. Everybody is sociable and they go out their way to make conversation and you don't feel unwelcomed and stuff like that so. It's just a really all round good experience to have. I would advise everybody if they had the chance to jump at it. It's a good opportunity."(P60:PI)

"Obviously, I've fair enjoyed it with Tia, my dog, I love spending time with her. But I think I'd be able to fulfil my role a bit better if I had more sessions where I'm purely helping other people. I'd be happy to do that, give a bit more of a hand to other people. I think if you have one session a week where you can just focus on your own dog, and then work as a peer mentor or assistant for the other sessions, it would be good."(P4:PMI)

"Because the paperwork that's getting done, I done that all on the last course, I've just been helping other boys. They're asking me for advice on what goes into the paper work and I'm just, not giving the answers but giving them advice on what to put in."(P37:PMI)

"It's better than when you first come up cos like, when you first come up you don't know what to do and you're not sure and stuff like that. But when I first came up and I wasn't sure, and people were telling us, 'do this, do this, do that' I was like, that's good man. Obviously I'm in that same position now. I can teach them and if they learn just one thing out of like 10 things I teach them then that's good."(P40:PMI)

**Communication skills**	*"Aye. I communicate with people better. So I'm happier doing it."(P66:PI)*<br>*"Aye. I've learned when to just calm down, man. Listen and pay attention, cos I like doing what I'm doing."(P46:PI)*
**Families**<br>Sharing experiences and skills with family; Parenting and child safety	*"Definitely. I was speaking to my mum about that actually and I was telling her what I've been doing here and she was like 'you can come out and train my dog' and stuff like that. Everything I've kind of learned in here is useful."(P60:PI)*<br>*"I do think it will affect me quite a lot. Cos most of my aunties and all that have got dogs so I'll show them that I've learned something from being in the jail doing this. That's what I want to do when I get out there, show people that I've not wasted my time being in the jail."(P33:PI)*<br>*"Aye, it helped me. It's helped me, 'cause I could tell my dad about it. Because we done it all... I made sure I really paid attention and then it gave me something to talk to them about."(P6:PI)*<br>*"My mam's really happy I'm doing this, that I'm sticking with it, she's fair proud of me."(P4:PMI)*<br>*"It's helpful for families too, and for weans, raising kids – you approach things differently, think about it as teaching – positive reinforcement. And it stops you just reacting to a situation – you take a step back and think about it instead."(P10:PI)*<br>*"It is very useful, if you've got weans in the house you know what signs to look for with the dogs."(P49:PI)*
**IMPULSIVITY**	
**Institutional behaviour**<br>Improved behaviour and relationships with staff and peers; Awareness of consequences	*"We are learning to be patient for the dogs for them to take to learning. This is also helping me in the hall cos I am more patient with staff."(P60:WS)*<br>*"Aye I've seen a difference… Sometimes in the hall and that I could be fighting another boy but I just thought like, if I do that I'll patch (leave) the dogs so I can't be bothered with it all."(P40:PI)*<br>*"Since I've been at Dog Training I think I've been calmer. Just like, I like coming up and I like working with the dogs, I like the dog that I've got. Just trying to be more calm in the hall and try and tone it down a wee bit. So I don't want to screw this up."(P40:PMI)*<br>*"After a couple of months at Polmont, I already had my fair share of reports, fights and solitary confinement, mostly due to my own anti-social behaviour. Once I'd been accepted onto the course I was told that I better change my behaviour or I'd risk being removed from it. After just one day on the course, having enjoyed myself so much, I decided to give it a go and make a real effort to change my ways. As the weeks went on I continued to work hard on improving my behaviour both on the training course and in general. I was actually beginning to surprise myself with how well I was doing. For the first time in my life I began putting others before myself and started thinking about the consequences of my actions before doing something. If I was put on report on the day before our training session I would then miss that training session, as would the dog I'd be working with. He would stay at the kennels while all the other dogs came in, so therefore he would effectively be punished for something I'd done. I didn't like the sound of that, and so thankfully I avoided that ever happening to me."(P8:WS)*
**Self-control**<br>Managing behaviour; Sense of achievement at progress	*"Paws for Progress has helped me stay calm and stay out of trouble."(P16:WS)*<br>*"Aye. Definitely useful. Now obviously I'll be able to control myself and work with it."(P55:PI)*

"Aye I've seen a difference cos usually I'm just like, dead impulsive. Like, if somebody says something to us, I'd say something back. But no, I just patch (leave) it now."(P40:PI)

"Usually I'm really impulsive and I don't think before I do something. But obviously I need to think before, for the dog's safety and before I do anything." (P40:PI)

EMOTIONAL MANAGEMENT

Patience

Appropriate pace for dogs to learn; Patience with other people; Benefits for self-development

"Patience. Obviously the dogs don't learn right away so I think I've got a wee bit more patient. You learn to slow down a wee bit."(P32:PI)

"I noticed I gained a lot more patience and I wasn't as quick and easy to judge others, you try to see their best instead. And I gained a lot more confidence in myself."(P5:PI)

"Aye. I feel like my patience and that is a lot better. I feel like I'm a lot more understanding of others."(P69:PI)

"I discovered that I was more patient than I thought I was. Eh, I could learn new skills if I just put my mind to it and thought about it."(P27:PI)

"I thought it's been very useful and that for us, because it gave us a lot more patience."(P69:PI)

"I also really enjoy working with the dogs as I did not have much patience before I came to the work with the dogs but now I would say my patience has really improved over the duration I have been here." (P30:WS)

Controlling anger

Importance for dog welfare; With other people; Benefits for self-development

"Aye. Dogs are like people. Because see when you get angry with a person you lash out. With like, a dog if they don't do as you're told - you don't want to lash out at a dog because you're like the controller. The trainer. Responsible. I had problems before – with anger and that – but I think I'm better managing it now." (P13:PI)

"The anger management side of things I would say… It's definitely helping my problem-solving skills a lot more."(P4:PI)

(Negative experiences)

Dog being rehomed; Negative situations commonly facing dogs; Past experiences of the dogs.

"Murphy was a wee bit of a challenge, I was sad to see him go and that, but obviously that's part of the course, what you're aiming for. That's the aim of the course anyway, to be getting them rehomed."(P4:PI)

"Learned about dogs getting put down. That was quite bad. Learned to be calm with dogs."(P48; PI)
